# Olfactory signals and fertility in olive baboons

**DOI:** 10.1038/s41598-021-87893-6

**Published:** 2021-04-19

**Authors:** Stefano Vaglio, Pamela Minicozzi, Sharon E. Kessler, David Walker, Joanna M. Setchell

**Affiliations:** 1 Department of Biology, Chemistry and Forensic Science, University of Wolverhampton, Wulfruna Street, Wolverhampton, WV1 1LY UK; 2 Department of Anthropology & Behaviour, Ecology and Evolution Research Centre, Durham University, South Road, Durham, DH1 3LE UK; 3 Cancer Survival Group, Department of Non-Communicable Disease Epidemiology, London School of Hygiene and Tropical Medicine, Keppel Street, London, WC1E 7HT UK; 4 Faculty of Natural Sciences, University of Stirling, Stirling, FK9 4LA Scotland, UK

**Keywords:** Biological techniques, Ecology, Evolution, Zoology

## Abstract

Female primates signal impending ovulation with a suite of sexual signals. Studies of these signals have focussed on visual, and to a lesser extent, acoustic signals, neglecting olfactory signals. We aimed to investigate the information content of female olfactory signals in captive olive baboons (*Papio anubis*) and relate these to the female fertile period. We studied eight adult females living in four groups at the CNRS Station de Primatologie, Rousset-sur-Arc, France. We used vaginal cytology to detect ovulation. We investigated the volatile component of odour signals using solid-phase microextraction and gas chromatography-mass spectrometry. We found a total of 74 volatile compounds, of which we tentatively identified 25, including several ketones, alcohols, aldehydes, terpenes, volatile fatty acids and hydrocarbons that have been identified in odour profiles of other primates. Our results show that vaginal odour intensity differs with sexual cycle stage suggesting that odour might play a role in signalling female baboon fertility. We found differences in vaginal odour between females living in all-female and in mixed sex groups but we could not distinguish the effects of group composition, female age and identity. This study of olfactory signalling improves our understanding of how female primates advertise their sexual receptivity.

## Introduction

Female primates are faced with a dilemma. They benefit from giving some males a higher probability of paternity, to obtain direct (e.g., access to resources, protection) or indirect (i.e., “good genes”) benefits from the “best” male^1^. However, they also benefit from confusing paternity, to reduce the risk of infanticide^2^. The “graded-signal” hypothesis suggests that females solve this dilemma by advertising their receptivity to attract and mate with multiple males (confusing paternity), while at the same time biasing the chances that preferred males sire the resulting offspring, by signalling the probability, but not the exact timing, of ovulation^3^. According to this hypothesis, female advertisement should be honest enough to give the preferred male a reasonable degree of paternity certainty, but should possess built-in unpredictability or error, which leaves other males with a smaller, but greater than zero, probability of sirehood^3^. Females thus manipulate male behaviour by altering the costs and benefits of mate-guarding, such that dominant males attempt to monopolise a female at the time when she is most likely to ovulate, while other males mate at sub-optimal times.

Female primates advertise impending ovulation with a variety of signals which are attractive to males, including some of the most conspicuous signals exhibited by mammals—sexual swellings^4^—as well as proceptive behaviour, copulation calls and olfactory signals^5–9^. Many studies have investigated the relationship between female sexual signals, the fertile period and male sexual behaviour in primates. These studies show that the degree to which males are able to assess a female’s reproductive status varies among species. For example, male sexual behaviour is closely related to female hormones and the fertile period in Tonkean macaques (*Macaca tonkeana*)^10^, long-tailed macaques (*Macaca fascicularis*)^11^, Barbary macaques (*Macaca sylvanus*)^12^ and Japanese macaques (*Macaca fuscata*)^13^, suggesting that males can accurately determine the female fertile period in these species. However, this is not the case in Hanuman langurs (*Semnopithecus entellus*)^14^, where male behaviour is unrelated to the timing of ovulation. This variation between species is not surprising and reflects different positions in an “ongoing battle of the sexes over paternity information”^15^.

While studies of female sexual swelling size, behaviour and vocalisations have accumulated^3,11,16–19^, olfactory communication has been neglected^20^, despite the fact that male primates clearly pay attention to female olfactory signals^21^. This is particularly the case for Afroeurasian monkeys and apes (catarrhines), which have been considered “microsmatic”^22,23^ (i.e., having a reduced olfactory sense^24^) with a simultaneous amplified emphasis on vision^25–27^. However, several studies suggest that odour may play an important role in catarrhines^28–38^, including the first detailed chemical analyses of scent-gland secretions for a non-human catarrhine—the mandrill (*Mandrillus sphinx*)^39,40^.

In non-human catarrhines, a systematic assessment of the possibility that olfactory cues are involved in the probability of ovulation in chacma baboons (*Papio ursinus*) concluded that odour represents one component of a multimodal signal of ovulation^41^. However, this study does not include chemical investigation of the olfactory signal. In non-catarrhine primates, contraceptives may disrupt intraspecific interactions in female ring-tailed lemurs (*Lemur catta*), by altering olfactory cues, including those relevant to kin recognition and mate choice^42^. There is also evidence from chemical analyses that odours encode information on fertility states in non-catarrhine primate species such as ring-tailed lemurs^43^ and common marmosets (*Callithrix jacchus*)^5^. Additionally, human observers have long noted strong-smelling vaginal secretions in female bonnet macaques (*Macaca radiata*) at mid-cycle^44^. These vaginal secretions are attractive to males, possibly due to volatile aliphatic acids, termed “copulins”^45,46^, which are also found in women’s vaginal secretions^47^. “Copulins” were thought to stimulate the mounting activity and ejaculatory behaviour of male rhesus monkeys, but other authors were unable to replicate these findings and attributed the males’ sexual responsiveness to the effects of associative learning^48^. Women’s scent varies significantly across the menstrual cycle^49^ and is more attractive to men during the late follicular phase (near ovulation) than in other cycle phases^50–52^. Finally, experiments on male stump-tailed macaques (*Macaca arctoides*) have shown that olfactory cues in vaginal discharges inform males as to female reproductive status^53^. Together, these studies support the hypothesis that female primates employ olfactory signals to advertise their sexual cycle status to males.

Features such as identity, rank, age and parity may influence the reproductive quality of female primates^54^, and may be reflected in their odour. In particular, it is widely hypothesized that every individual has their own unique body odour, like a fingerprint^55^. Moreover, rank (e.g., common marmosets^56^) and age [e.g., owl monkeys (*Aotus *spp.)]^57^ affect the scent of female primates. However, the effect of parity on odour has not yet been studied.

Although the role of olfaction in female sexual communication has been almost entirely neglected in catarrhines, olfactory signals have clear potential to convey information concerning female fertility and are of obvious interest to males (see^58^ for a review of female sexual signals, including sexual swellings, facial colour and odour, in mandrills, and related implications for on-going theoretical debates in the field of sexual selection). In this context, olive baboons (*Papio anubis*) are an excellent model species as they live in multi-male, multi-female groups in which females mate polyandrously. Several studies of female signalling exist for baboons^19,59^, but no studies have investigated chemical signalling in this species, although it is clear that males investigate female vaginal secretions and thus olfactory communication may play a crucial role in advertising female sexual receptivity.

Odour is linked directly to physiological condition and is therefore expected to be more honest than other types of signal^60^. Like humans, baboons are catarrhines, and share the loss of olfactory genes and the reduction in the reliance on olfaction that occurred during catarrhine evolution. If this study of baboons reveals that olfactory cues in catarrhines play similar roles to those as found in primates that are more distantly related to humans, this would provide further evidence that viewing anthropoid primates as “microsmatic” is premature.

In this study we combined the chemical investigation of vaginal odour with the timing of the fertile period, estimated by using cytological criteria, to evaluate cycle-dependent changes in female olfactory signals and their reliability as indicators of the fertile phase in captive female baboons. The use of a captive colony provided the opportunity to study the relationships between semiochemistry and cytology in detail.

The overarching aim of the study was to test the explanatory power of the graded-signal hypothesis for olfactory signals for the first time. According to this hypothesis, dominant males tend to guard females only at peak swelling and, therefore, females could use vaginal odour signals to advertise the exact timing of ovulation only to preferred males who have close access to their genitalia during mate-guarding behaviour. We thus predicted clear and consistent changes in vaginal olfactory signals associated with the females’ fertile window. We examined variation in odour signals across the female cycle and related this to the probability of ovulation, and described the variation in the dataset and highlighted promising areas of future research. In particular, we aimed to:

1. Describe the chemical composition of female vaginal odour and identify compounds that may be of interest for future work.
2. Describe variation in female odour across cycle phases.
3. Conduct preliminary tests of whether females differ in their odour cues by age/group composition within a cycle phase.
4. Conduct preliminary tests of whether, within an age-group class, female odour differs by cycle phase.
5. Conduct preliminary investigations of whether female odour is individually distinctive in the non-fertile phase.

## Methods

### Study subjects

We studied olive baboons living at the Station de Primatologie, Centre National de la Recherche Scientifique (CNRS), Rousset sur Arc (France). We observed sexual swellings and sampled genital cytology and vaginal odour for the same 12 unrelated females, all with regular menstrual cycles and aged 4.5–24.4 years at the beginning of the study. Wild female olive baboons experience menarche at four years^61^, are considered sexually mature at 4.5 years old^62^ and first conceive at approximately five years old^63^. Colony-reared baboons show menarche up to a year earlier and a slightly shortened time between menarche and first conception^63^. Unpublished data from the Station de Primatologie confirmed that females reach sexual maturity around 3.5 years in this captive population (Romain Lacoste, personal communication). We collated each female’s reproductive history (i.e., births, and age at parturition) from colony records.

We collected data for four months, following 38 cycles for six females housed in three small neighbouring groups consisting of six females and one male each (we sampled two females per group) and six females in a larger all-female group (20 females). In the context of this study, we focused on a dataset including eight females, each of which contributed 2–4 cycles (total: 25), with four females housed in the all-female group and four females housed in one-male multi-female groups (Table 1). The four females housed in the all-female group were nulliparous and younger (4.6–5.3 years) (*group type FF hereafter*). The four females living in male–female groups were parous and older (10.5–24.4 years) (*group type MF hereafter*). All males were vasectomised; this should not affect their sexual behaviour since this procedure does not remove the gonads, which remain active. All baboons had equal access to the same quality of food items (i.e., monkey biscuits, plus cultivated fruits during the sampling sessions) meaning that we can exclude any dietary impact on the chemical compounds excreted by the baboons.

**Table 1:** Study subjects at the beginning of data collection.

Female name/identity code	Female age at the beginning of the study (years)	Group composition/identity code	Number of sexual cycles	Number of odour samples
Solene/968	12.6	MF/B1.4	3	20
Tulie/1128	11	MF/B1.5	3	15
Talka/4	11	MF/B1.5	3	14
Plutea/955	15.1	MF/B1.6	3	20
Delphine/1237	5.3	FF/B8	4	48
Elodie/1349	5	FF/B8	3	20
Epura/1399	4.6	FF/B8	2	9
Elsa/1376	4.6	FF/B8	4	36

The dataset was reduced from 12 to eight female baboons in order to preserve vaginal odour samples for future work.

Before data collection, we used positive reinforcement training^64^ for 10 weeks to train subjects to present their sexual swelling to us and allow us to collect vaginal swabs for cytology and secretions for odour sampling.

### Reproductive parameters

#### Morphological changes in the anogenital area

During the follicular phase of the menstrual cycle, the olive baboon anogenital area increases in size due to estrogenic stimulation^21^. Ovulation occurs most commonly during the last few days of maximal swelling, frequently two days before the swelling subsides^65–68^.

**Figure 1:**
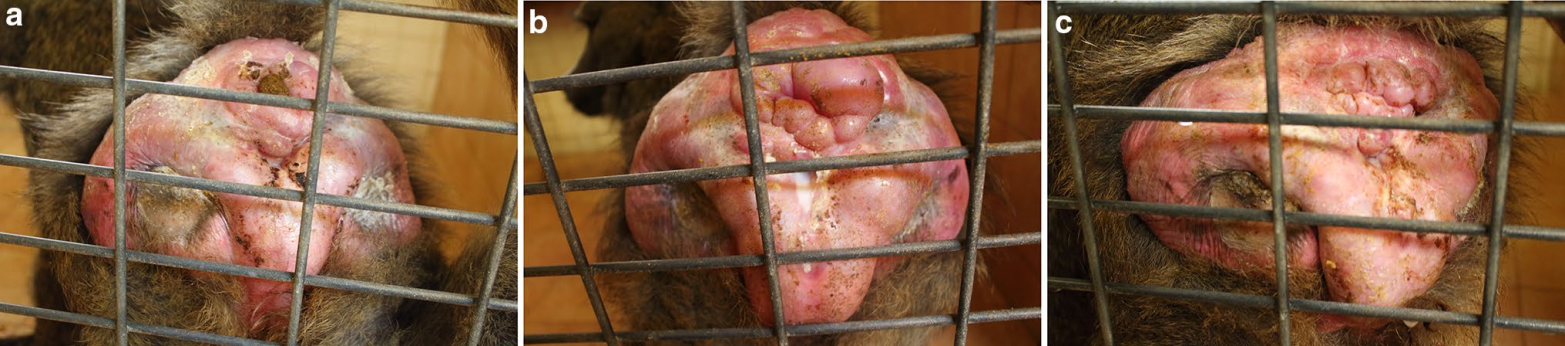
Pictures of moderate (**a**), large (**b**) and deflating (**c**) genital swellings of the same female baboon across her menstrual cycle.

We made daily records of morphological changes in the anogenital area and menstruation 6 days per week (Monday to Saturday). We noted anogenital area characteristics as: menstruation; postmenstrual flat (immediately following menstruation, anogenital area is flat and faded); moderate genital swelling (reddening and partial tumescence of the anogenital area); large genital swelling (tumescence at maximum volume and bright pink); deflating genital swelling (the appearance of wrinkles, reduction of visible tumescence and loss of coloration); full detumescence (no tumescence and pale) (Fig. 1).

#### Genital cytology

We evaluated slides from days with moderate, large and deflating swellings using genital cytology to detect the ovulation window. Cytological evaluation of vaginal cells provides a reliable determination of the exact stage of the baboon menstrual cycle^63^.

We collected vaginal swabs from study subjects five days per week, Monday to Friday (we were not allowed to collect samples during weekends by the Station de Primatologie management team due to lack of keeper presence and related safety issues). We collected a full series of slides over the period with moderate, large and deflating swellings for 31 of 38 cycles. For the remaining seven cycles, we missed one sampling day during the large swelling period, when ovulation is most likely^69^.

To collect samples, we gently inserted a cotton-tipped swab inside the vulva, then rotated the end through 3 revolutions, to pick up sufficient vaginal cells for cytological evaluation, before gently withdrawing the swab. We prepared the smear immediately by rolling the cotton tip along the length of a glass microscope slide, and fixed it using a spray fixative (CytoRAL)^69^.

We prepared and stained vaginal smear slides using commercially available kits (RAL Diagnostics) at the Laboratory of Molecular Biology (Station de Primatologie - CNRS), and evaluated slides using LAS (4.3) software in the Department of Biosciences, Durham University. The RAL Diagnoestrus kit is a commercial simplified Harris-Schorr technique for use with vaginal smears. It consists of three rinsing solutions and involves an accurate procedure lasting around 16 min. We stained the smear following the kit protocol, dried the slide and applied a coverslip^69^.

**Figure 2:**
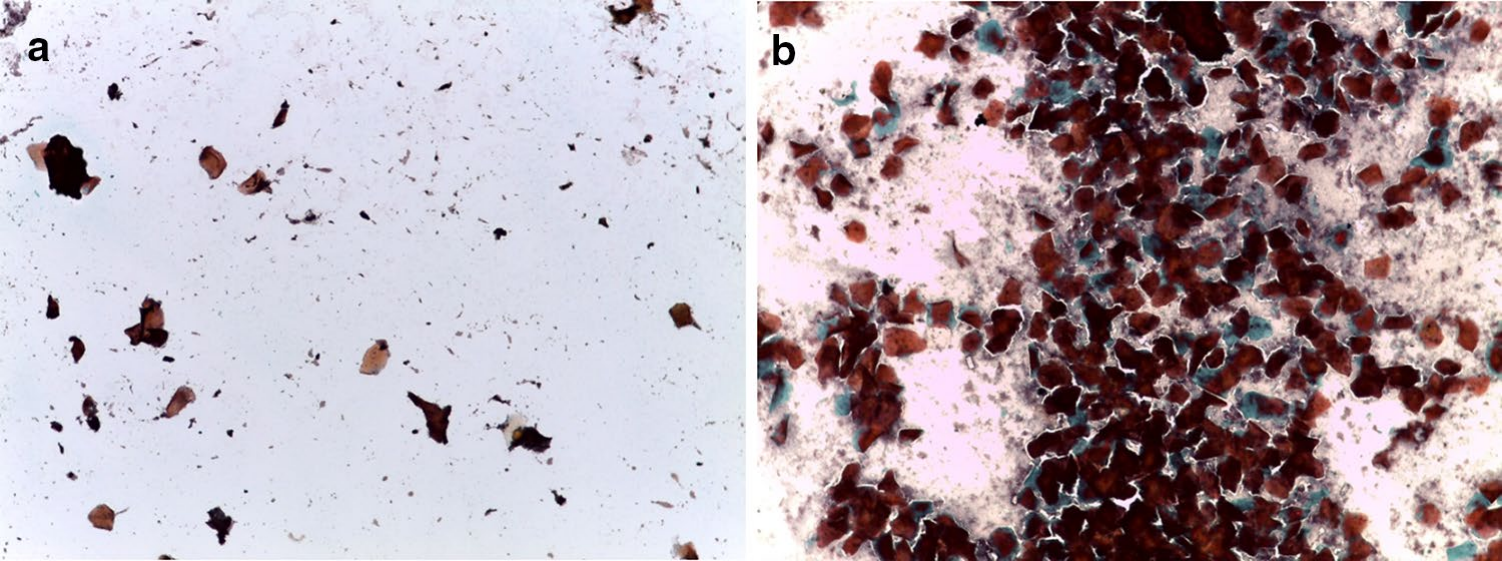
Genital cytology data confirming that females experienced regular receptive cycles. In comparison to the pre-ovulatory phase (**a**), the post-ovulatory phase (**b**) is marked by the return of leukocytes and mucus, as well as clumped, curled and folded cells and, quite commonly, placard or rosette arrangements of cells.

Hendrickx and Kraemer^66^ give an extensive description of the cyclical changes in vaginal epithelial cells through the baboon menstrual cycle. Honoré and Tardif^63^ and Shambayati^70^ describe the different cell types (superficial, intermediate, parabasal, and basal) characteristic of each phase in detail. We detected ovulation based on a sudden decrease in superficial cells with brownish tan granular cytoplasm and, in many instances, by intermenstrual bleeding^69^. The postovulatory phase is marked by the return of leukocytes and mucus, as well as clumped, curled and folded cells and, quite commonly, placard or rosette arrangements of cells (Fig. 2).

We considered the ovulation window as the day of ovulation and the previous day (31 cycles with full series of slides available) and the two days (remaining 7 cycles without full series of slides available) before vaginal cell populations changed abruptly.

#### Cycle phases

We considered the five day fertile phase as the two days covering the ovulation window and the three preceding days, to account for the lifespan of sperm in the female tract (humans^71^), following^12^. We labelled the five days preceding the fertile phase as the pre-fertile phase and the five days following the fertile phase as the post-fertile phase. We labelled the rest of the cycle as non-fertile.

### Odour sampling and analysis

We collected daily vaginal odour samples by rubbing sterile cotton swabs around the wall of the vulva 10 times, using steady pressure and a standardized protocol to standardize the amount of secretion we collected. In addition, we exposed control swabs to the air in the indoor enclosure during sampling to identify any chemical compounds that did not derive from the female baboons. We placed all samples and controls into sterile 10 ml screw-capped clear glass vials (Supelco thread: 18O.D. 22.5 mm × H 46 mm) closed by teflon-faced rubber septa and seals (1.3 mm thick). We stored vials at − 80 °C.

We transferred the samples on dry ice to the Rosalind Franklin Science Centre, University of Wolverhampton (UK), where we conducted the laboratory analyses. We investigated the volatile components of vaginal odour secretions using established solid-phase microextraction (SPME) and gas chromatography-mass spectrometry (GC–MS) and applying the same methods used in our previous work on lemur and mandrill odour signals [reviewed in^72^].

We introduced a 65 µm polydimethylsiloxane/divinylbenzene SPME syringe needle through the vial septum and exposed the fibre to the headspace above the sample in the vial for 15 min at 40 °C. We analysed the adsorbed volatile analytes of all samples using a 5975C mass spectrometer (Agilent Technologies) EI, 70 eV, coupled directly to a 7890B gas chromatograph (Agilent Technologies) equipped with a fused silica HP5-MS UI capillary column (Agilent Technologies) 30 m × 0.25 mm crossbonded 5%-phenyl-95%-dimethylpolysiloxane, film thickness 0.25 µm. We maintained the injector and transfer line temperatures at 270 °C and 280 °C, respectively. We made injections in splitless mode (purge valve opened after 1 min) with a constant flow of helium carrier gas of 1 ml min^−1^. We started the oven temperature program at 45 °C for 2 min, then raised it by 4 °C min^−1^ to 170 °C, and finally by 20 °C min^−1^ to 300 °C^40^.

We assessed possible environmental contamination via blank analyses using an empty 10 ml vial (Supelco) and control swabs following the same procedure as for the samples and conditioned the fibre at 260 °C pre-injection for 5 min and 260 °C post-injection for 20 min to avoid any possible carry-over effects.

We standardized peak retention times using retention time locking to alpha pinene. We tentatively identified eluted compounds by comparing the experimental spectra with those of the mass-spectral library in ChemStation (Agilent Technologies) and NIST Database (National Institute of Standards and Technology), version MSD F.01.01.2317 (Agilent Technologies). We accepted a putative identification if the minimum matching factor was higher than 90%. To minimize the chance of misidentification and when more than one compound was a good match for the same GC peak, we considered the chromatographic retention time and compared it with those reported in the literature for the same chromatographic column type^73^. We created a data matrix using the peak area relative to each identified compound using the integrated signal of the deconvoluted total ion current (TIC). We analysed all samples in a short period of time to minimize interassay variability. We overlaid chemical profiles from control swabs on animal chemical profiles to identify compounds that did not derive from the animals and removed these from the swab results.

### Statistical analyses

#### Data structure

Our dataset had a complex structure, with eight females contributing 3–5 cycles (total: 27). Not all cycles contained samples for each cycle phase (non-fertile, pre-fertile, fertile, post-fertile). We excluded samples taken while the female was menstruating. The nature of the sample, with four young females housed in an all-female group and four older females housed in bisexual groups, made it difficult to isolate the effects of age, group type (FF vs. MF), group, individual, and cycle stage. Moreover, samples taken at time points close together (i.e., consecutive days or within the same phase of the same cycle) may be more similar than samples taken further apart (i.e., days or months apart, or from the same phase of *different* cycles), making them pseudoreplicates. Where possible, we control for these effects using subsets of the data, but we regard our findings as preliminary, and they should be confirmed in future work.

We used two analytical approaches—one uses a subset of the samples but all compounds; the other uses all samples but only the most abundant compounds. These two sets of statistical analyses are alternative approaches needed due to the complexity of the data set.

#### Calculating variables and testing assumptions

The raw data consisted of a matrix of the signal intensities which represent relative quantities of each compound in the odour profile of each sample. We calculated the variable Total Odour as the total area under the curve for each sample which represents the summed intensity across the entire range of masses detected at every point in the analysis. To compare changes in the relative compositions of odour profiles across samples, we calculated the percentage contribution of each compound to the total odour for that sample. We used a heat map to visualize patterns of compound abundance (Fig. S1). We also calculated three diversity indices for each sample: Richness (total number of compounds present in the sample), Simpson’s D (D = 1/(Σp_i_^2^)), and Shannon’s H (H = − Σp_i_lnp_i_), where p_*i*_ is the proportion of compound *i* relative to the total number of compounds. In contrast to Richness, Simpson’s D and Shannon’s H incorporate information about the relative abundances of the compounds, or evenness, of the chemical profiles.

The dataset contained many low abundance compounds (median number of samples a compound was detected in: 28.5, range: 6–117, n = 148). To handle this, we used nonmetric multidimensional scaling to reduce the dataset of 74 compounds (coded as percentages) to two new variables. We used the function metaMDS in the package vegan v.2.5–5^74^ (R 3.6.0^75^ together with RStudio 1.2.1335^76^). The procedure used Euclidean distances and a maximum of 1000 random starts when searching for a stable solution. Although similar in aim, this procedure is not exactly analogous to a nonparametric principal components analysis so there are no component loadings to report. Due to the sparseness of the dataset for the FF females, the procedure did not converge when we included them. Thus, we ran metaMDS on data from the MF females only. We tested whether the two multidimensional scaling output variables (MDS1 and MDS2) were correlated using Spearman’s Rank Correlation.

For the first analytical approach, we tested for violations of normality and differences in variances by group type (FF vs. MF), and fertility phase (comparing fertile vs. non-fertile phases) (Tables S1–S4). Because of violations of normality and equal variances, we use nonparametric statistical methods whenever possible. We used parametric statistical methods for the second analytical approach (as detailed below). We set alpha at 0.05. We considered p ≤ 0.1 as a trend. We use Bonferroni corrections for multiple testing when applicable. We ran statistics in SPSS 25 (IBM Statistics), using R 3.6.0^75^ together with RStudio 1.2.1335^76^), and Stata^77^.

#### Selecting a subset of matched pairs

To reduce the effects of pseudoreplication within a cycle, we selected a smaller dataset of matched sample pairs, with pairs matched by cycle. First, we selected a sample from the fertile phase from each cycle, as close to the date of ovulation as possible. Then we selected a corresponding non-fertile sample where the cycle stage was recorded as ‘full detumescence’. We selected the closest eligible sample in time, going either backwards or forward. In two cycles, no non-fertile sample met our criteria, so we used a non-fertile sample from the end of that female’s previous cycle. For one additional cycle the non-fertile sample is from 8.5 weeks prior (i.e., two cycles) earlier than the fertile sample. Only one cycle was sampled twice for non-fertile samples. This gave us 15 matched pairs of fertile/non-fertile stage samples. While this smaller dataset eliminates pseudoreplication within a cycle, it does still include pseudoreplication within individual females because some females contributed up to 3 cycles. We tested this matched-pairs dataset for correlations between total odour and compound richness using Spearman’s rank correlation test. For comparison, we present results conducted on a reduced dataset of one sample (the first in the dataset) for each female, providing a total of one pair of samples (fertile/non-fertile, matched by cycle) per female in the Supplementary Files.

#### Differences between FF and MF females

Using the subset of 15 cycles, we tested for differences between the MF and FF females using Mann Whitney U tests for Total Odour, Richness, Simpson’s D, and Shannon’s H. Because Simpson’s D and Shannon’s H are both diversity measures of evenness, we applied a Bonferroni correction across these tests (test-wide alpha = 0.05). We ran separate tests on the fertile and non-fertile phases. We tested whether the FF females could be discriminated from the MF females with a nested pDFA^78^ on the non-fertile phase data using Total Odour and Richness. This procedure uses a function (provided by R. Mundry) based on the function lda of the R package MASS^79^. We used group type (MF vs. FF) as the test factor and Subject ID as the control factor (eight subjects) with no restriction factor, 1000 random selections, and 10,000 permutations. We included 78 samples, and used two samples from each individual to derive the discriminant functions. We cross-classified the remaining samples. We did not have enough samples to test for differences in the fertile phase.

#### Comparison between fertile and non-fertile phases

We tested for differences between fertile and non-fertile phases in Total Odour, Richness, Simpson’s D, and Shannon’s H using Wilcoxon Matched Pairs tests on the matched pairs dataset of 15 cycles. We split the dataset by group type (MF: 7 cycles, FF: 8 cycles) and ran separate tests on each group type. As above, we applied a Bonferroni correction across Simpson’s D and Shannon’s H (test-wide alpha = 0.05).

We used a repeated measures MANOVA on MDS1 and MDS2 to test for differences between fertile and non-fertile phases in the MF females (data matched by 7 cycles). Although this is a parametric test, we are not aware of a suitable test which is both nonparametric and accounts for repeated measures.

We repeated all the analyses using generalised linear mixed models (GLMM)^80^ with Total Odour, Richness, Simpson’s D, Shannon’s H or individual compounds as the outcome variable, individual baboon as a random effect, and individual cycle and social group as predictors with fixed effects. Either identity or log scales were used, according to the outcome distribution. No random slopes were included in the models. For the analyses with individual compounds as the outcome, we focused on the 15 compounds with measurements above the third quartile of the distribution for FF and MF separately, to reach the minimum sample size required for analysis^81^. All models were visually inspected for normal distribution of all levels of residuals. The homoscedasticity assumption was relaxed by specifying an unstructured covariance matrix. To achieve more reliable P values, we fitted GLMMs using maximum likelihood (rather than restricted maximum likelihood). The significance of full models was tested using likelihood ratio tests. We used Bonferroni’s method to adjust for multiple pairwise comparisons across the levels of considered factor variables.

#### Individual differences

We ran a series of pDFAs^78^ testing whether we could differentiate between females based on their odours in the non-fertile phases. (i) We tested whether three FF females could be discriminated based on their total odour and compound richness. We excluded one of the original four females because her samples contained insufficient variation for the pDFA to run. We excluded three cycles with only one non-fertile sample to comply with the requirement that there should not be more variables than cases in the smallest level. This left 42 samples encompassing three individuals and nine cycles. We ran nested pDFAs with cycle as the control factor nested within individual (test factor). We selected three samples from each cycle to derive the discriminant functions and cross-classified the remaining cycles. (ii) We ran nested pDFAs testing whether the older MF subjects could be discriminated using (a) total odour and compound richness and (b) the two variables produced by the multidimensional scaling (MDS1 and MDS2). Subject ID was the test factor and cycle was the control factor. The dataset had 34 samples spread across four subjects, who together had a total of 8 cycles. We selected two samples per cycle to create the discriminant functions and cross-classified remaining samples. All pDFAs by individual used 1000 random selections and 10,000 permutations. Although we are testing for differences between individuals, we cannot fully disentangle individual differences from group differences for the MF females.

### Ethical statement

We used non-invasive techniques. The study protocols were approved by the Ethics Committees of the Department of Anthropology, Durham University, Durham (UK) and of the Station de Primatologie, Centre National de la Recherche Scientifique, Rousset sur Arc (France). We confirm that the study was performed in accordance with the Directive 2010/63/EU of the European Parliament and of the Council of 22 September 2010 on the protection of animals used for scientific purposes.

## Results

### Odour secretions

**Figure 3:**
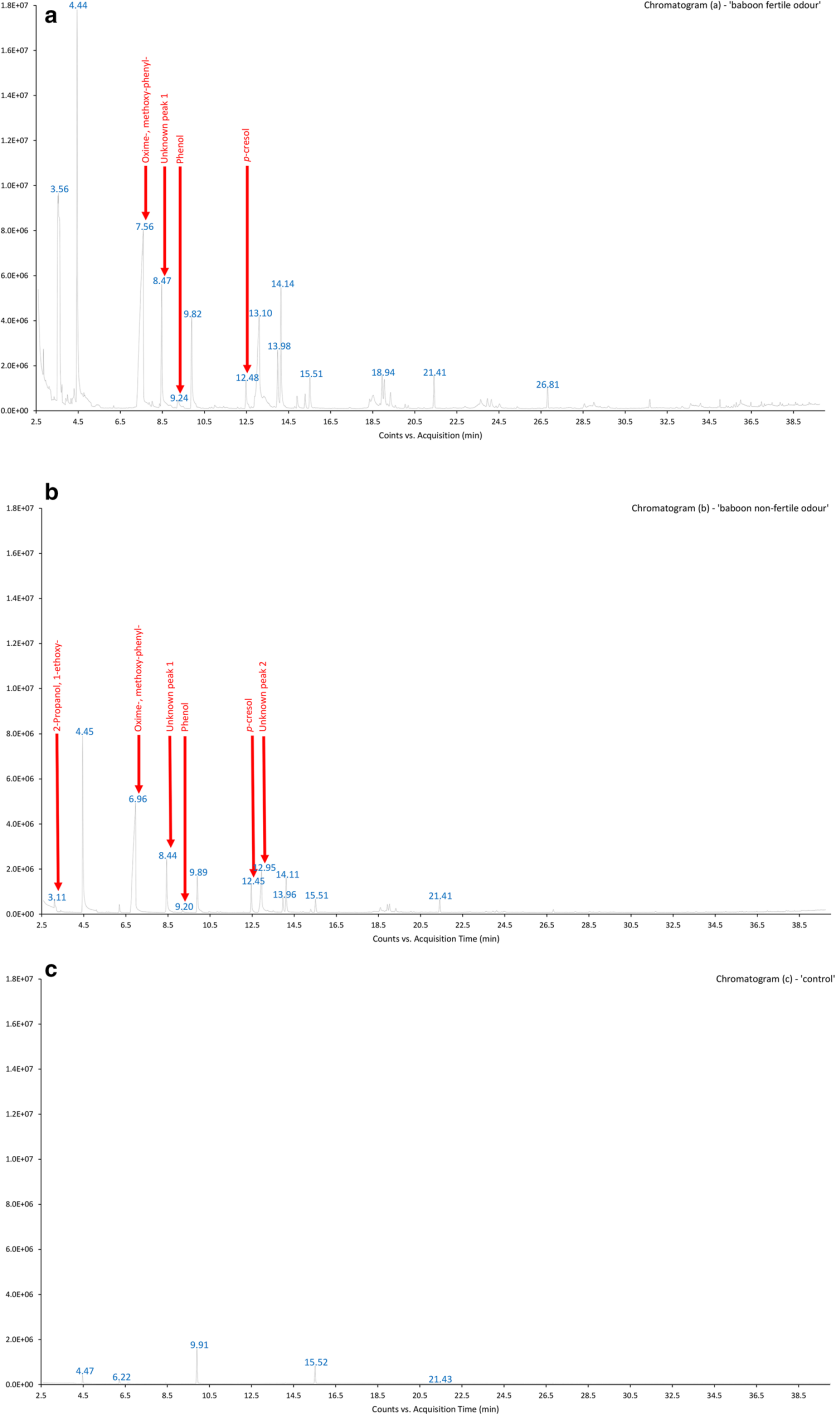
Example chromatograms from female olive baboon, vaginal odour sample from fertile period (chromatogram (**a**)—‘baboon fertile odour’); female olive baboon, vaginal odour sample from non-fertile period (chromatogram (**b**)—‘baboon non-fertile odour’); and control sample, showing contaminants (chromatogram (**c**)—‘control’).

We found a total of 74 volatile compounds from the analysis of 182 vaginal odour samples. These compounds included a range of naturally occurring odorous volatile compounds such as ketones, alcohols, aldehydes, terpenes, volatile fatty acids and hydrocarbons. We could tentatively identify 25 compounds (Table 2), and 49 compounds were classified as “unknowns”. Figure 3 shows typical chromatograms used to compare control and female baboon vaginal odour samples from fertile and non-fertile periods.

**Table 2:** Volatile compounds present in swab samples from baboon vaginal odour secretions identified tentatively using the ChemStation (Agilent Technologies) and NIST (version MSD F.01.01.2317) mass spectral databases, listed in order of retention time.

Retention time (min)	Molecular weight (Da)	Compound	Primate species with same compound having semiochemical role
2.93	100.089	Methyl isobutyl ketone	Aye-aye, red-ruffed lemur
3.01	93.991	Disulfide, dimethyl-	Humans
3.08	104.084	2-Propanol, 1-ethoxy-	
3.23	88.052	Propanoic acid, 2-methyl-	
3.34	92.063	Toluene	Ring-tailed lemur, mandrill
3.78	88.052	Butyric acid	Common marmoset, humans
3.94	100.089	Hexanal	Aye-aye, red-ruffed lemur, ring-tailed lemur
5.38	270.256	Hexadecanoic acid, 2-methyl-	
5.39	106.078	Benzene, ethyl-	Red-ruffed lemur, mandrill
5.60	106.078	*m*-Xylene	Ring-tailed lemur, mandrill
6.20	104.063	Styrene	Owl monkey
6.70 to 7.50^a^	151.063	**Oxime-, methoxy-phenyl-**	Humans
7.23	120.094	Benzene, (1-methylethyl)-	Ring-tailed lemur, mandrill
8.16	120.094	Benzene, propyl-	Ring-tailed lemur, mandrill
8.36	106.042	Benzaldehyde	Aye-aye, red-ruffed lemur, common marmoset, emperor tamarin, Weddell’s saddleback tamarin, capuchin monkey, owl monkey, mandrill
9.10	128.120	1-Octen-3-ol	Ring-tailed lemur, Coquerel’s sifaka
9.20	94.042	Phenol	Aye-aye, red-ruffed lemur, hamadryas baboon, mandrill
9.40	170.203	Heptane, 2,2,4,6,6-pentamethyl-	Ring-tailed lemur, mandrill
10.74	136.125	D-Limonene	Red-ruffed lemur
12.45	108.058	***p*-Cresol**	Red-ruffed lemur, capuchin monkey
15.84	122.037	Benzoic acid	Coquerel’s sifaka, common marmoset
16.53	154.136	*α*-Terpineol	Red-ruffed lemur
16.88	152.120	2-Cyclohexen-1-ol, 3-methyl-6-(1-methylethenyl)-	
17.52	152.120	*cis*-Carveol	
20.03	117.058	Indole	

The compounds in bold font were found with highest abundance and most change from sample to sample.

^a^Peak overloading resulting in broad peak and RT variation.

### Statistical results

Many of the 74 compounds were only present in small amounts in a small number of samples (Figs. S1, S2a–j). The compounds with highest abundance and most change from sample to sample were methoxy-phenyl-oxime (RT 6.70 to 7.50), *p*-cresol (RT 12.45) and two unknown compounds (RT 8.43 and RT 12.84).

**Figure 4:**
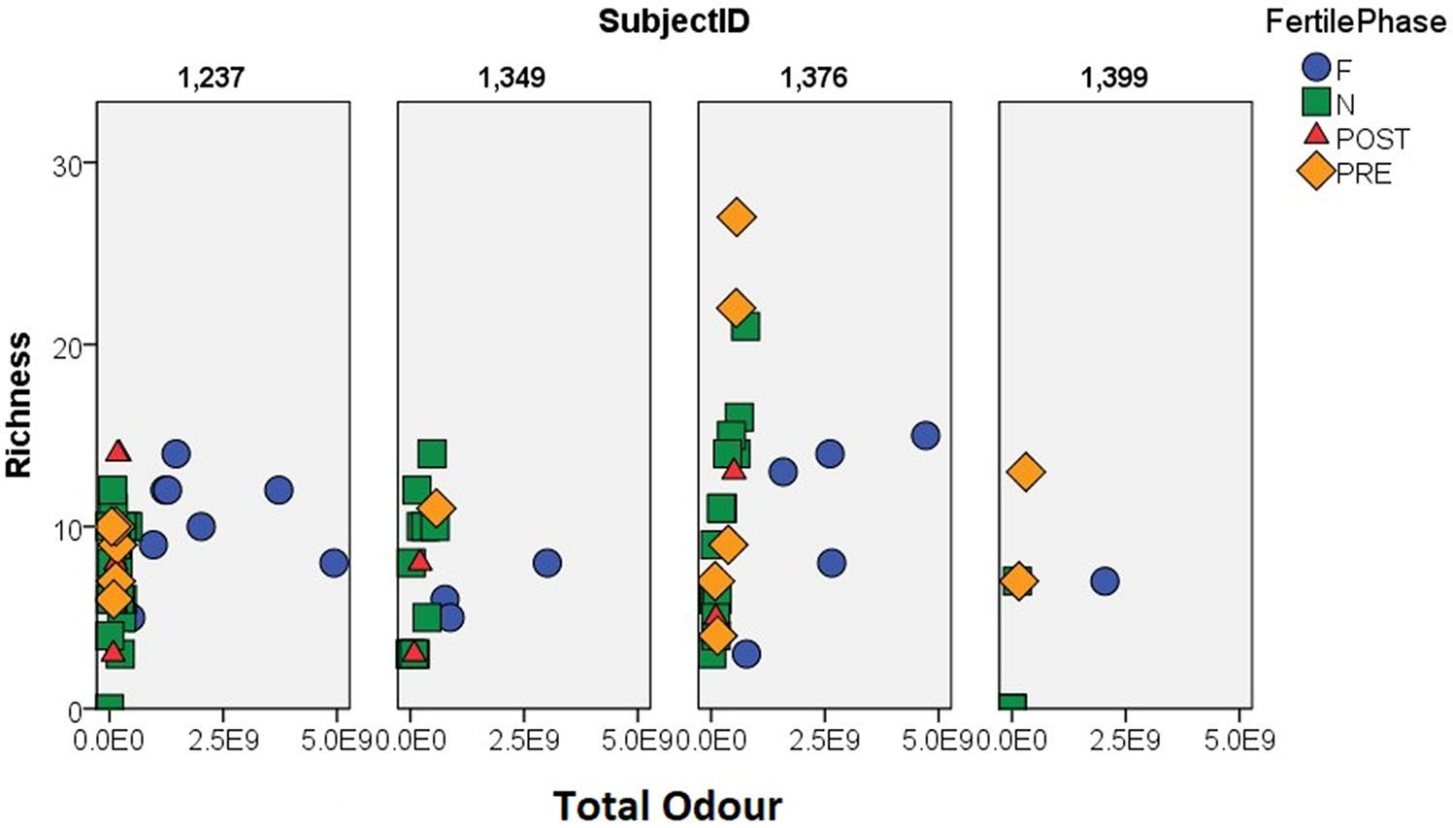
Scatterplots of Total Odour (x-axis) and Richness (y-axis) for each female, showing how the samples differ by fertility phase. The top row shows the FF females. The bottom row shows the MF females. Note that the Richness scale is smaller for the FF females. For both groups, Total Odour separates the fertile phase samples from the rest. F, N, Post, and Pre are fertile, non-fertile, post-fertile, and pre-fertile, respectively. FF indicates that the females are from an all-female group. MF indicates that the females are from a group with both sexes.

**Figure 5:**
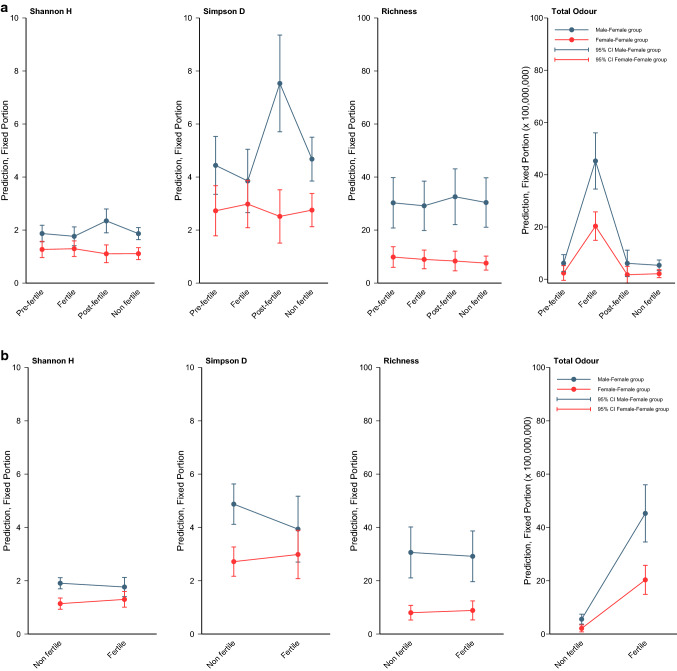
Predictions of the three diversity indices and of Total Odour by using GLMM estimates of the fixed effects.

**Figure 6:**
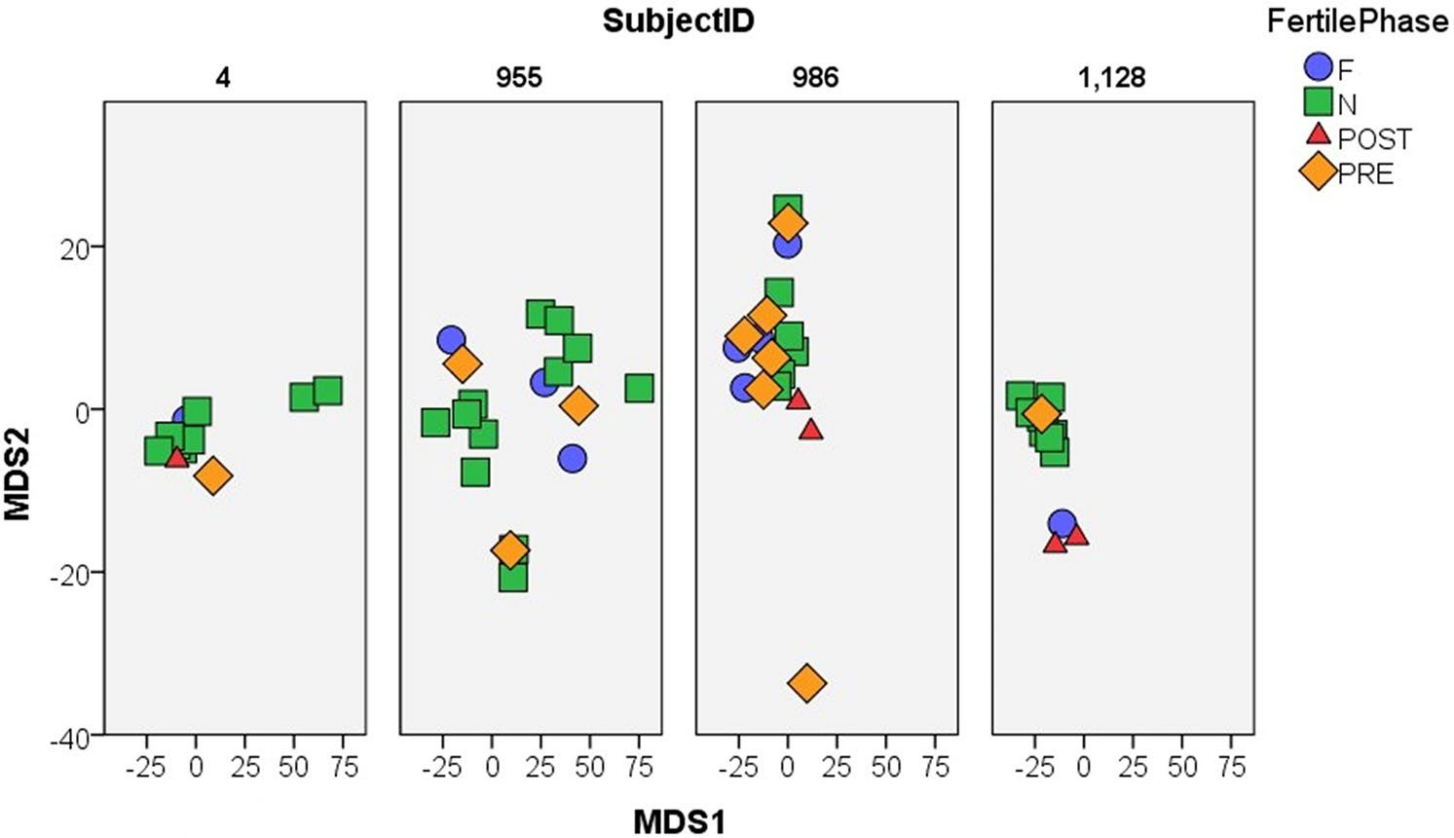
Scatterplot showing that MDS1 and MDS2 calculated from the percentage data do not separate the different cycle stages into clusters. F, N, Post, and Pre are fertile, non-fertile, post-fertile, and pre-fertile, respectively.

Total Odour and Richness values were very similar for samples collected during the non-fertile, pre-fertile, and post-fertile phases for all females (Fig. 4). For all females, fertile phase samples have noticeably greater Total Odour, but are within the Richness range of the other samples. These results were confirmed by the GLMM analyses (Fig. 5). Richness and Total Odour were significantly correlated in the matched pairs dataset of 15 cycles (r_s_ = 0.388, N = 30, p = 0.034). The two dimensions extracted from the multidimensional scaling procedure conducted on the MF females (MDS1 and MDS2) did not separate the samples into clusters by cycle stage (Fig. 6), nor were the two variables correlated (r_s_ = − 0.200, N = 14, p = 0.493).

**Figure 7:**
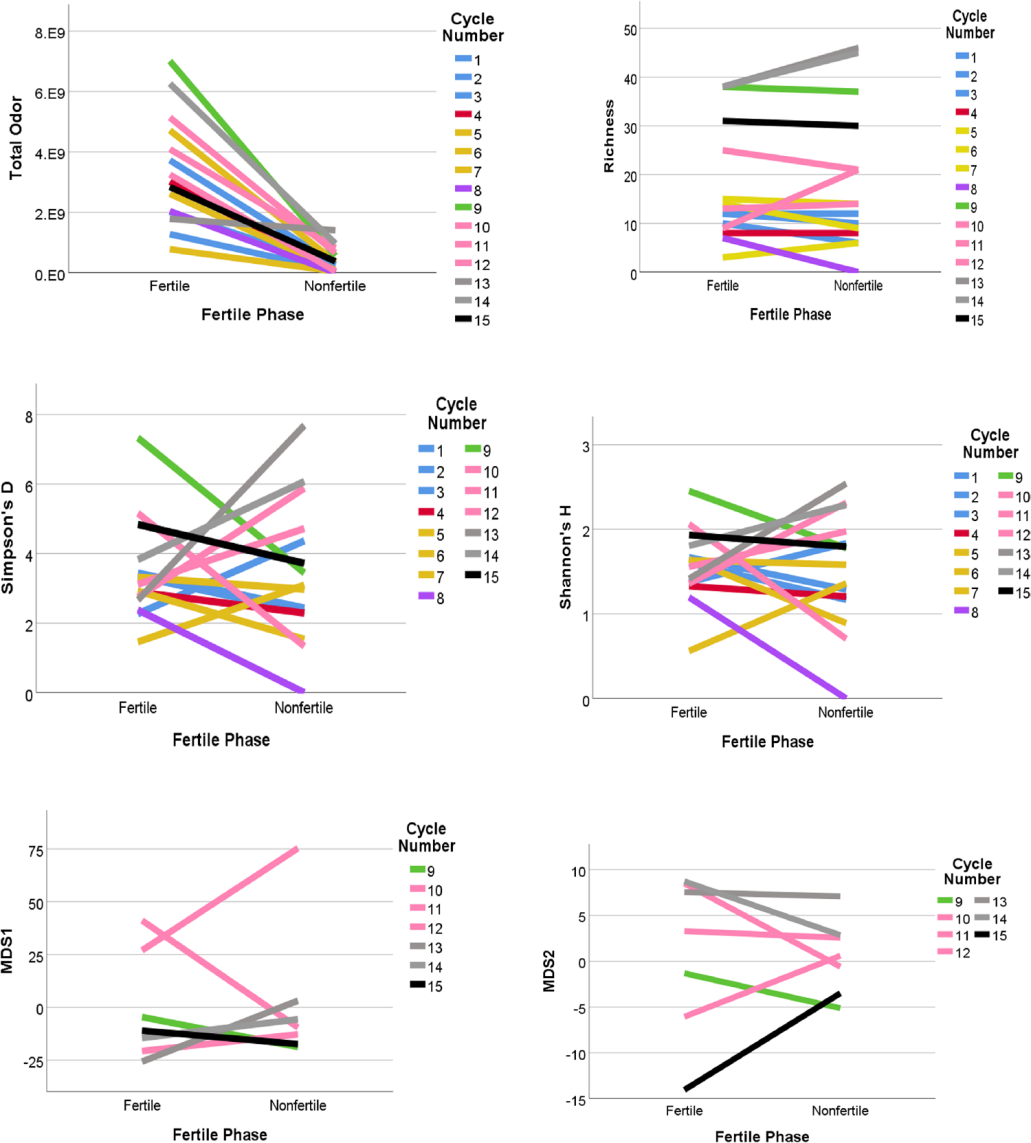
Comparison of the matched pairs of fertile and non-fertile samples for the 15 cycles, using Total Odour, Richness, Shannon’s H, Simpson’s D, and MDS1 and MDS2. Each line represents a cycle and the colours represent the different females. Only Total Odour shows a clear difference between non-fertile and fertile samples. Greater clustering of lines by individual (colour) indicates lower variability in the odour profile of each female.

Only Total Odour showed a clear difference between the non-fertile and fertile phase samples, with an increase in Total Odour during the fertile phase (Figs. 5, 7). The similarities and differences between paired samples for the same individual allow a visual evaluation of the variability of odour profile for each female. Samples from the same individual do not appear to cluster together.

#### Differences between younger, FF females and older, MF females

Considering both the matched pairs dataset and the whole dataset, MF subjects had greater compound richness in both the non-fertile and fertile phases than FF subjects (Table 3, Figs. 4, 5). MF total odour was significantly greater than in FF subjects in the non-fertile phase and showed a similar trend in the fertile phase (Table 3). Although Simpson’s D and Shannon’s H did not differ between the two groups in either phase after we applied a Bonferroni correction in the matched pairs dataset, these two diversity indices differed between MF and FF during the non-fertile phases (Table 3). For results using one cycle per female or four categories for individual cycle, see Table S5. Total Odour and Richness provided sufficient information for the samples to be significantly classified by group type (MF vs. FF: pDFA, correctly cross-classified: 89%, expected to be correctly cross-classified: 60%, p = 0.034).

**Table 3:** Evaluation of the differences between MF and FF subjects.

Variable	Mann Whitney U tests				Pairwise comparisons			
	Non-fertile		Fertile		Non-fertile		Fertile	
	U	P	U	P	T	P	T	P
Total Odor	5	**0.008**	12	0.064	− 2.99	**0.006**	− 4.06	**< 0.001**
Richness	0.5	**0.001**	7	**0.015**	− 4.46	**< 0.001**	− 3.92	**< 0.001**
Simpson’s D	9	0.028*	13	0.083	− 4.52	**< 0.001**	− 1.22	0.447
Shannon’s H	9	0.028*	13	0.083	− 5.02	**< 0.001**	− 1.97	0.098

Results from Mann Whitney U tests (all tests were conducted on 7 MF cycles and 8 FF cycles**) and pairwise comparisons (tests conducted on the whole datasets after having fitted GLMMs using two categories for individual cycle**).

Bolded p-values are significant.

*Not significant after a Bonferroni correction (adjusted alpha < 0.025).

**For results using one cycle per female or four categories for individual cycle, see Supplementary Table S5.

#### Comparison between fertile and non-fertile stages

Considering both the matched pairs dataset and the whole dataset, Total Odour was significantly greater in the fertile phase than the non-fertile phase for both the FF and the MF females (Table 4). Richness, Simpson’s D, and Shannon’s H did not differ between fertility phases. Results using one cycle per female, which have less power, or four categories for individual cycle, show the same trends, but they are not statistically significant (Table S6). Fertile phase did not have a significant effect on MDS1 and MDS2 using the 15 cycle dataset (repeated measures MANOVA, Pillai’s Trace = 0.012, F(2, 5) = 0.031, p = 0.969).

**Table 4:** Evaluation of the differences between fertile phase and non-fertile phase.

Variable	FF		MF		FF		MF	
	Z	P	Z	P	T	P	T	P
Total Odor	− 2.521	**0.012**	− 2.366	**0.018**	6.93	**< 0.001**	8.00	**< 0.001**
Richness	− 1.572	0.116	− 1.022	0**.**307	0.70	0.481	− 0.89	0.376
Simpson’s D	− 0.700	0.484	− 0.338	0.735	0.58	0.559	− 1.52	0.129
Shannon’s H	− 0.840	0.401	− 0.338	0.735	1.25	0.209	− 0.82	0.412

Results from Wilcoxon Matched Pairs tests (tests conducted with 7 MF cycles and 8 FF cycles*) and pairwise comparisons (tests conducted on the whole datasets after having fitted GLMMs using two categories for individual cycle*).

Bolded p-values are significant.

*For results using one cycle per female or four categories for individual cycle, see Supplementary Table S6.

**Figure 8:**
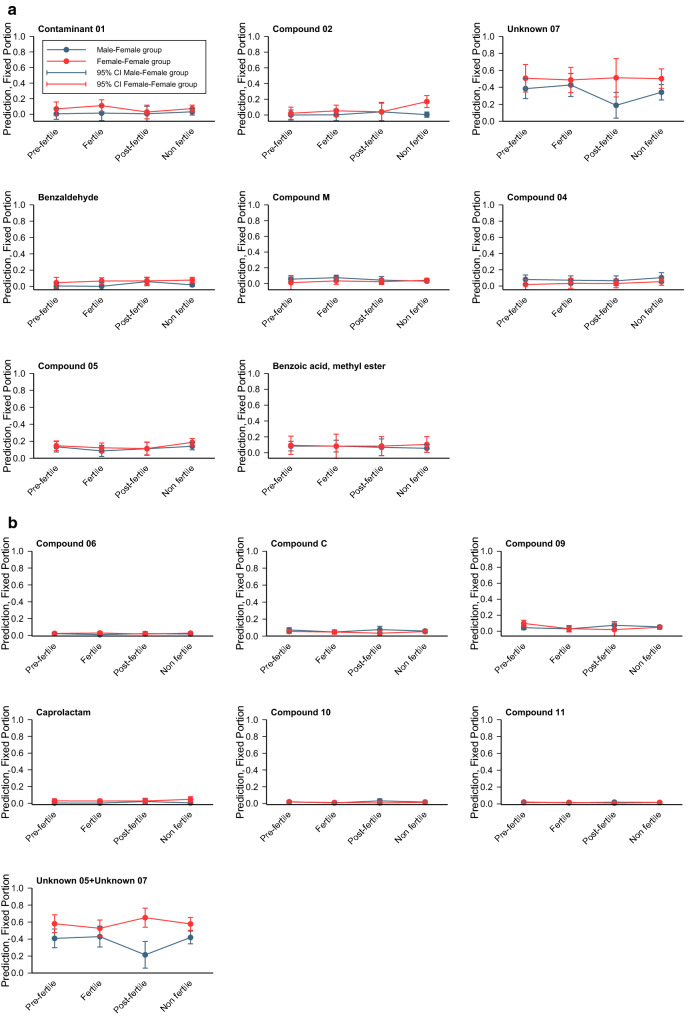
Predictions among the 15 individual compounds with measurements above the third quartile of the distribution for FF and MF separately by using GLMMs. No compounds differed significantly between fertile and non-fertile phases.

Among the 15 individual compounds with measurements above the third quartile of the distribution for FF and MF, separately, no compounds differed significantly between fertile and non-fertile phases (Fig. 8, Table S7).

Total Odour and Richness were able to correctly classify 89% cross-classified FF samples by fertility phase, which showed a non-significant trend (pDFA, expected to be correctly cross-classified: 62%, p = 0.068). The classification accuracy for the MF samples was even higher, with 97% of the cross-classified calls being correctly classified, but this was not statistically significant (pDFA, expected to be correctly cross-classified: 66%, p = 0.114). MDS1 and MDS2 were not able to accurately classify the MF samples by fertile phase (pDFA, correctly cross-classified: 44%, expected to be correctly cross-classified: 50%, p = 0.663).

#### Individual differences in the non-fertile phase

The FF females were less individually distinct than the MF females. The three FF females could not be accurately discriminated using Total Odour and Richness (pDFA, correctly cross-classified: 29%, expected to be correctly cross-classified: 39%, p = 0.815). In contrast, the MF females could be discriminated using Total Odour and Richness (pDFA, correctly cross-classified: 75%, expected to be correctly cross-classified: 51%, p = 0.036) and showed a trend towards being distinguishable using MDS1 and MDS2 (pDFA, correctly cross-classified: 48%, expected to be correctly cross-classified: 32%, p = 0.1). However, we were unable to statistically distinguish whether these differences among the MF females stem from individual differences or differences between their groups (as the four MF females come from three different MF groups).

## Discussion

Very little is known about the chemical changes underpinning the olfactory cues of reproductive quality in primates, due to methodological challenges in sampling, recording and quantifying volatile chemical profiles of odours^82^. We used positive reinforcement training to collect high quality vaginal swabs for cytology and secretions for odour sampling, combined with an effective methodology for extraction and analysis of chemical signals^72^. We described the chemical composition of female vaginal odour and identified compounds that may be of interest for future work; we then investigated the variation in female vaginal odour across cycle phases and explored the possible role played by age, parity, identity and group composition. However, we could not investigate parity and age as individual factors due to our study setting and, more generally, our preliminary findings should be confirmed in future research work.

We found a total of 74 volatile compounds in female baboon vaginal odour secretions but tentatively identified only 25 (excluding environmental and lab contaminants as well as co-eluted compounds). We found both low-molecular-weight, highly volatile compounds and high-molecular-weight, less volatile compounds. In particular, we found volatile hydrocarbons (such as toluene, ethyl benzene, 1,3-dimethyl benzene (*m*-xylene), 1-methylethyl benzene, propyl benzene, and 2,2,4,6,6-pentamethyl heptane) which have previously been identified in odorants deriving from ring-tailed lemurs (*Lemur catta*), red-ruffed lemurs (*Varecia variegate rubra*) and Coquerel’s sifakas (*Propithecus verreauxi coquereli*)^83–85^ as well as from catarrhines such as mandrills^39,40^ (see Table 2 for details). In addition, high-molecular-weight, less volatile hydrocarbons, such as several unknown hydrocarbons found in this study, may act as a fixative which slows the release of more volatile compounds, as seen in house mice (*Mus musculus*)^86^ and cotton-top tamarins (*Saguinus oedipus*)^87^.

Volatile fatty acids contribute to human body odour deriving from the apocrine sweat glands^88^. Among the acids (2-methyl propanoic acid, butyric acid, 2-methyl hexadecanoic acid, and benzoic acid), we found the compound butyric acid that is one of the five aliphatic acids, named “copulins”, which were also found in women’s vaginal secretions and considered to contribute to making these vaginal secretions attractive to males^89^. Likewise, methoxy-phenyl-oxime was identified in human axillary sweat^90^, and reported as a minor constituent of essential oils and aromatic extracts^91^; conversely, the other compound which we found with highest abundance and most change from sample to sample, *p*-cresol, has previously been identified in female scent-marks by red-ruffed lemurs^84^ and capuchin monkeys^92^ as well as a component of the female sex pheromone in the horse (*Equus caballus*). Additionally, the compounds benzoic acid, hexanal and 1-Octen-3-ol are encountered in odour secretions used to scent-mark by several non-primate mammals (e.g., lions (*Panthera leo*), African wild dogs (*Lycaon pictus*), gray wolves (*Canis lupus*), house mice (*Mus musculus*), red foxes (*Vulpes vulpes*)) (reviewed in^93^) and primates (e.g., aye-ayes (*Daubentonia madagascariensis*)^94^, red-ruffed lemurs^84^, ring-tailed lemurs^43^, Coquerel’s sifakas^83^, common marmosets^95^) (see Table 2 for details). The compound benzaldehyde is considered a crucial putative semiochemical occurring at all ancestral nodes leading to both urine and glandular markers in many strepsirrhine species^96^; this compound has also been found in scent gland secretions released by ayes-ayes^94^, red-ruffed lemurs^84^, common marmosets^95^, capuchin monkeys^92^, emperor tamarins^97^, Weddell’s saddleback tamarins^97^, owl monkeys (where the presence of this compound has also been validated with internal standards)^57^ and mandrills^39,40^, and acts as a sex pheromone in other mammals and as an alarm pheromone in invertebrates such as stingless bees (*Tetragonisca angustula*) (reviewed in^73^). Interestingly, methyl ketones, which are found as putative semiochemicals in aye-ayes^94^ and red-ruffed lemurs^84^, dominate females’ skin pheromone blend in garter snakes (*Thamnophis sirtalis parietalis*), and their presence and relative abundance are used by males to choose female mates with potential for production of more offspring per litter^98^. Furthermore, volatile dimethyl disulphide was identified as an odorous compound resulting from diseases caused by infectious bacteria, such as cholera, in humans (reviewed in^99^), while phenol has been found in vaginal odour secretions of several primate species (e.g., ayes-ayes^94^, red-ruffed lemurs^84^, hamadryas baboons (*Papio hamadryas*)^100^) and also serves as the locust phase change pheromone produced by gut bacteria^101^. Finally, indole is released as an anti-aphrodisiac during mating by male butterflies (*Pieris rapae*)^102^, whereas D-limonene derives from leaves and flowers and is used by male insects (e.g., euglossine orchid bees (*Euglossini *spp.)) to display successfully and attract females^103^.

We described variation in female vaginal odour across cycle phases. We found that total odour and compound richness were very similar for female baboons during their non-fertile, pre-fertile and post-fertile phases, while all females showed significantly greater total odour during the fertile phase (but within the richness range of the other phases). In particular, total odour was significantly greater during the fertile phase than during the non-fertile phase for both FF and MF subjects; however, Richness, Simpson’s D, and Shannon’s H did not differ between fertility phases. Total odour showed a clear difference between the non-fertile and fertile phases, with a significant increase in values during the fertile phase.

Our findings support the hypothesis that female baboons use olfactory signals to advertise their reproductive status to males, and that, in particular, females use odour intensity to advertise the precise timing of their fertile window to preferred males; i.e., dominant males performing mate-guarding. These males can evaluate the visual signals (i.e., changes in size and shape of female sexual swellings^104^) and monopolise females at the time when they are most likely to ovulate, while other males will mate at sub-optimal times. These results also suggest that odour signals allow females to control the information available to individual males. While a visual or acoustic signal is broadcast to all males present, it is likely that only dominant males will be able to approach and smell a female’s genitalia^105,106^. Therefore, an accurate indication of the probability of ovulation may be available only to dominant males. Hence, as suggested by the “graded-signal” hypothesis^3^, females may be able to solve their dilemma by offering different male audiences different information about their reproductive status, thus confusing and biasing paternity at the same time.

When comparing MF and FF females, MF females had greater compound richness in both the non-fertile and fertile phases. MF females also showed a significantly greater total odour in the non-fertile phase and a similar trend in the fertile phase. The occurrence of extra compounds in the vaginal odour secretions released by MF females might relate to the presence of group males; i.e., scent mixing as males deposit sperm-related compounds in the female’s vagina and on her vulva. This could mean that males can advertise their presence and features (e.g., sex, rank, age, health status) to other males (i.e., both subordinate group males and external males) inspecting the female vaginal odour. Several species, such as ring-tailed lemurs^43^, use composite olfactory signals that incorporate odorants from multiple sources to increase information content (‘multiple-messages’ hypothesis)^107^ or prolong signal longevity (‘fixative’ hypothesis)^108^, even when the scent signal delivery is ‘passive’ (i.e., as reflected by the natural diffusion of odours from specific regions of the body).

MF females also had higher richness than FF females in the non-fertile phase; i.e., when they would not be mating. Since group type (FF females vs. MF females) is confounded with age (FF females are younger than MF females), another potential explanation for the patterns we found is that the differences in compound richness is due to the age difference between FF and MF subjects. This interpretation is consistent with evidence that body odours carry age-related information and that an individual’s age can be determined by its scent in several animal species (reviewed in^109^). The chemical composition of body odours changes in an age-dependent manner in several non-human animals, such as house mice^110^, otter (*Lutra lutra*)^111^, night monkeys (*Aotus nancymaae*)^112^, and humans^109^; additionally, both non-human animals, such as house mice^113^ and giant pandas (*Ailuropoda melanoleuca*)^114^, and humans^109^ can infer the age of conspecifics based on body odours. It is crucial for animals to inform conspecifics about their age. For instance, age may correlate with parity. Older reproductive females, who are also expert mothers, may be more likely to conceive and succeed in raising their offspring^115^; in contrast, younger females could be on the cusp of sexual maturity and so might have lower conception rates than older females^21^. Moreover, younger females, who are still investing in their own somatic growth, may have smaller offspring^115^.

Odour signals are crucial in competitive interactions between female mammals (reviewed in^116^). Female odour may provide reliable signals of competitive ability to both female competitors and potential male mates. Older reproductive females, therefore, might develop odour signals to outcompete younger female individuals for access to the best mates. This hypothesis is also supported by the high costs of mate-guarding behaviour displayed by male baboons^117^, who invest high levels of energy and time to monopolise the most attractive females despite living in large multi-male, multi-female groups in which females mate polyandrously.

The odour of FF females was less individually distinctive than that of MF females. FF females could not be accurately discriminated using total odour and compound richness. In contrast, MF females could be discriminated using total odour and richness and showed a trend towards being distinguishable using MDS1 and MDS2. However, individual is confounded with group; i.e., the four MF subjects are spread across three groups, while all four FF subjects live in one group. The only reason to expect systematic odour differences across such neighbouring MF groups is the presence of different males; i.e., mating behaviour. This would support the hypothesis that males ‘allomark’ females with their own odour, advertising their presence and features to other males. Alternatively, these odour differences might be due to individual differences, rather than group differences. Future studies could test this hypothesis by focusing on larger groups of baboons with comparable group composition; i.e., where group and individual identity are not confounded.

Mammalian social systems rely on signals passed between individuals conveying a variety of information, including individual and group identity. In particular, an individual odour signature is made by multiple sources of chemosignals, based on both non-volatile (i.e., proteins) and volatile compounds (reviewed in^118^). For example, odour may encode information about signaller identity in several mammal species^119,120^, including primates such as lemurs^43–60,73,82–89,93,96,98,99,104–122^, marmosets^73,84–89,93,95,96,98,99,104–122^ and mandrills^39^; furthermore, lemurs^122^, various South American monkeys^95,123,124^, and humans^28^ can discriminate between the scents of individual conspecifics. We found that identity may play a role in advertising female sexual receptivity to males, but further work is needed to understand such role and ensure that is not a group effect.

## Conclusion

In conclusion, this study of olfactory signalling shows that baboon vaginal odour may contain information about sexual cycle status suggesting that odour plays a role in signalling the timing of the fertile period. We also found differences in vaginal odour between group types but we could not distinguish the effects of group composition, female identity, age and parity. These findings contribute to improving our understanding of how female non-human primates advertise their sexual receptivity.

Future research work should validate the identity of vaginal odour compounds by using certified reference standards. Additionally, as vaginal odour intensity could be a simple by-product of hormone modulation or microbial action (i.e., not necessarily part of female advertisement) and its differences across female cycle phases not necessarily salient to males, future work should examine information perceived by the recipient; for instance, testing the male response to female odours via bioassays. Another hypothesis that could be tested in the future would be whether females become smellier when they mature; i.e., producing greater individual differences between older reproductive females than younger adolescent females. Furthermore, as olfactory signals have the potential to communicate both genetic quality and genetic compatibility to prospective mates, it would be interesting to investigate whether major histocompatibility complex genotype is encoded in female genital odour in primates.

Lastly, as the study of human chemical communication benefits from comparative perspectives with non-human primates and as olive baboons are an excellent model species for comparison to humans, this study is pertinent to olfactory communication in humans. In particular, further work could resolve questions of interest for humans, such as whether and how olfaction is related to fertility, via detailed chemical analyses of odour secretions in different body regions across the menstrual cycle, which could be addressed using a similar methodological approach.

## Supplementary Information

Supplementary Information 1.

Supplementary Figure S2.

## Data Availability

The raw data are available on request.
