# Supplementary material for: Olfactory signals and fertility in olive baboons
Source: Sci Rep. 2021 Apr 19;11:8506. doi: 10.1038/s41598-021-87893-6 (PMC8055877; doi:10.1038/s41598-021-87893-6)
Supplement: Supplementary file 2 — Supplementary Figure S2. [file 41598_2021_87893_MOESM2_ESM.zip › Figures S2a-j/FigureS2b.pdf]

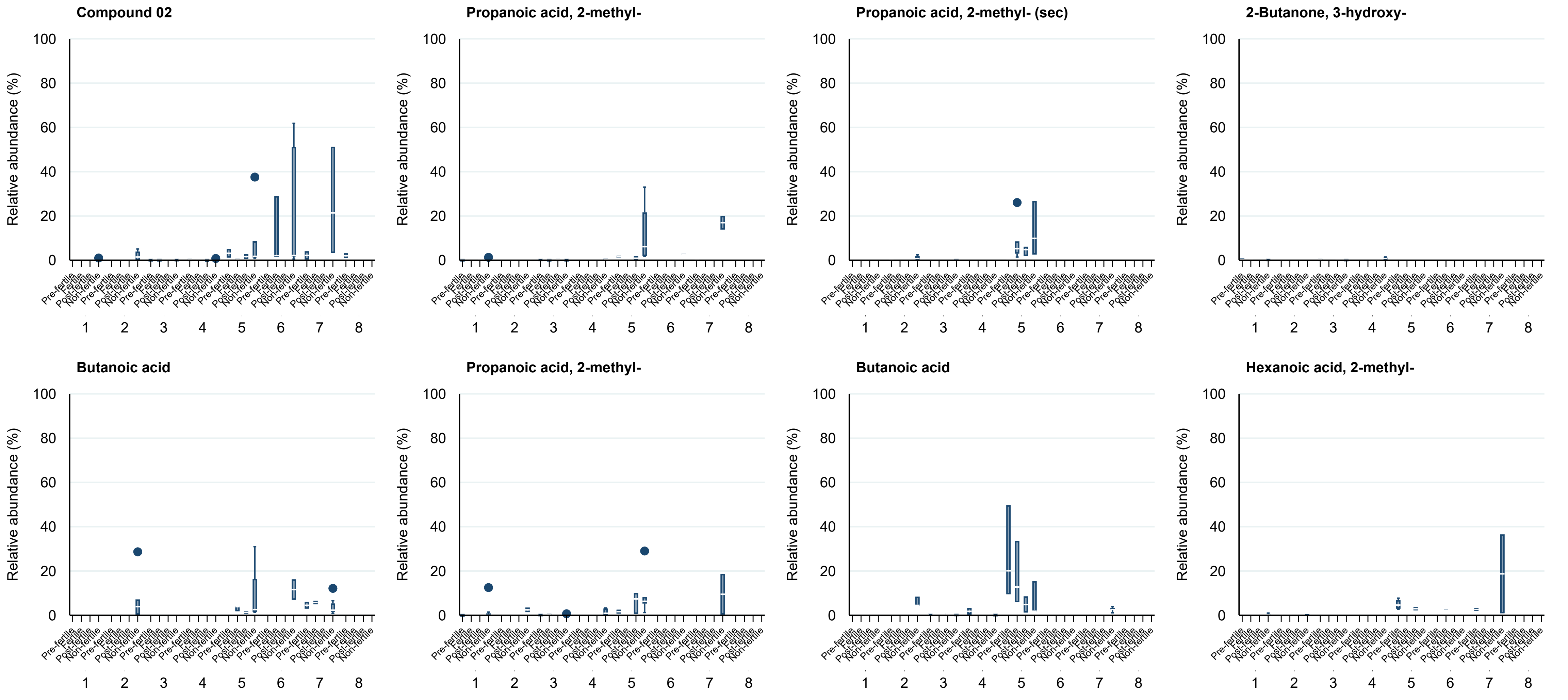
Compound 02
Propanoic acid, 2-methyl-
Propanoic acid, 2-methyl- (sec)
2-Butanone, 3-hydroxy-
Butanoic acid
Propanoic acid, 2-methyl-
Butanoic acid
Hexanoic acid, 2-methyl-
Relative abundance (%)
0
20
40
60
80
100
Pre-fertile
Fertile
Post-fertile
Non-fertile
1
2
3
4
5
6
7
8
