# Supplementary material for: Olfactory signals and fertility in olive baboons
Source: Sci Rep. 2021 Apr 19;11:8506. doi: 10.1038/s41598-021-87893-6 (PMC8055877; doi:10.1038/s41598-021-87893-6)
Supplement: Supplementary file 2 — Supplementary Figure S2. [file 41598_2021_87893_MOESM2_ESM.zip › Figures S2a-j/FigureS2c.pdf]

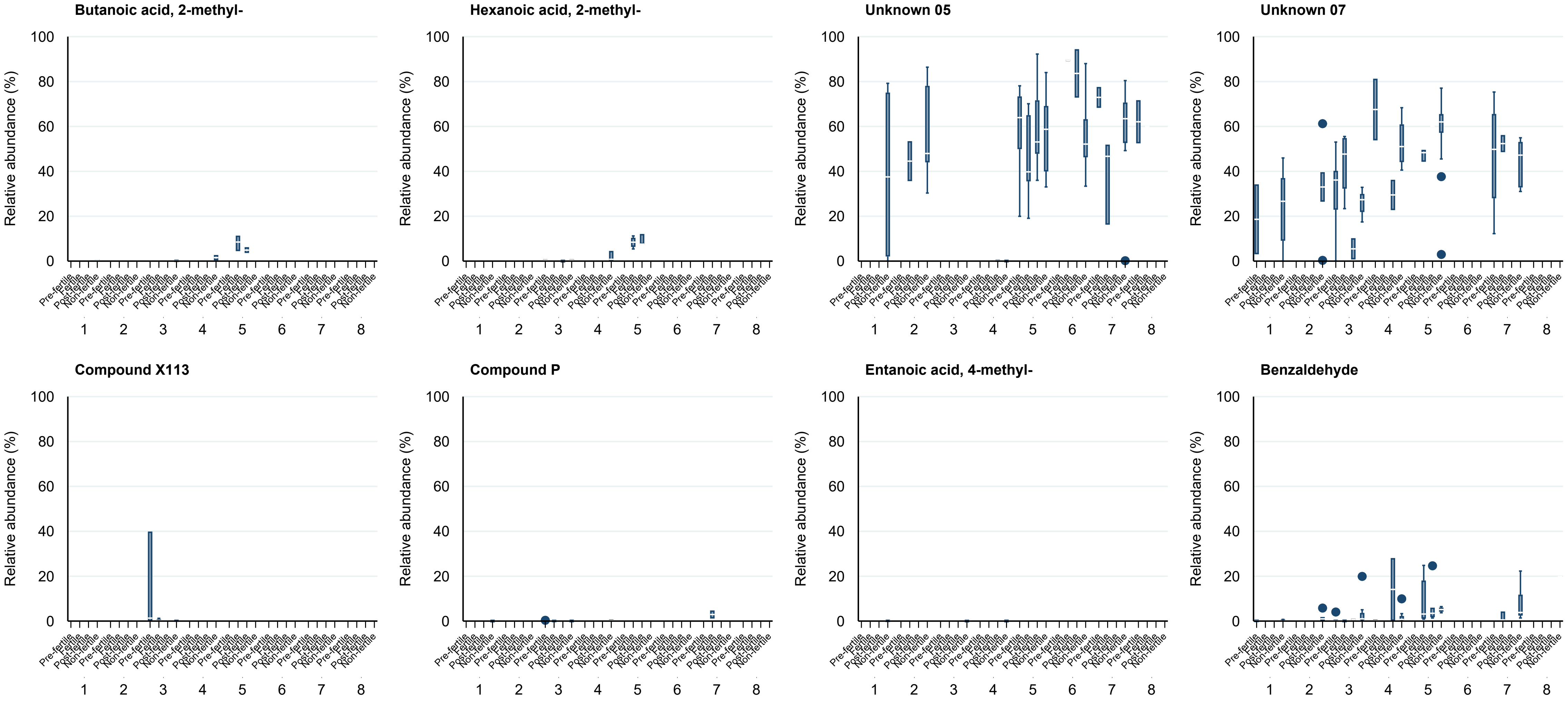

Butanoic acid, 2-methyl-
Hexanoic acid, 2-methyl-
Unknown 05
Unknown 07
Compound X113
Compound P
Entanoic acid, 4-methyl-
Benzaldehyde
Relative abundance (%)
0
20
40
60
80
100
Pre-fertile
Fertile
Post-fertile
Non-fertile
1
2
3
4
5
6
7
8
