# Supplementary figures and images for: Olfactory signals and fertility in olive baboons
Source: Sci Rep. 2021 Apr 19;11:8506. doi: 10.1038/s41598-021-87893-6 (PMC8055877; doi:10.1038/s41598-021-87893-6)

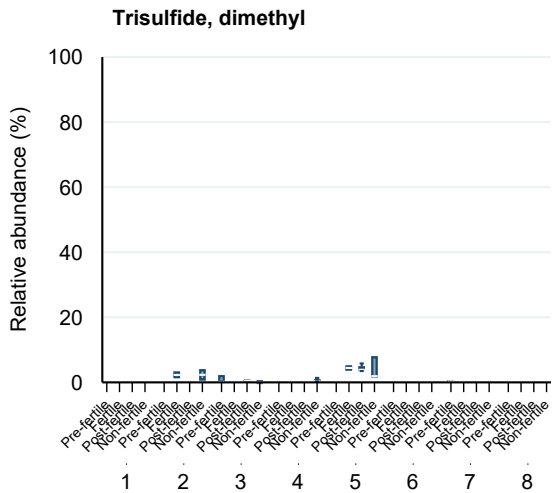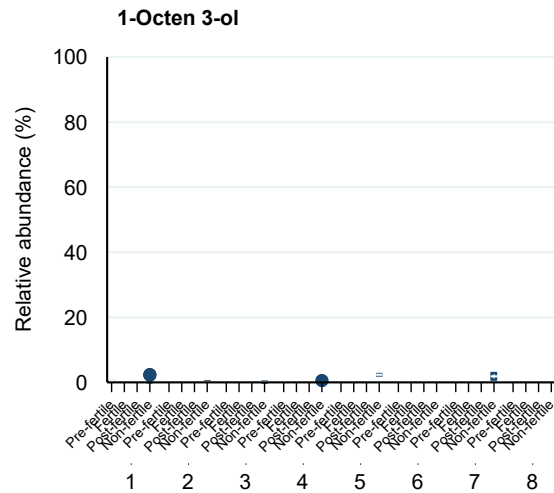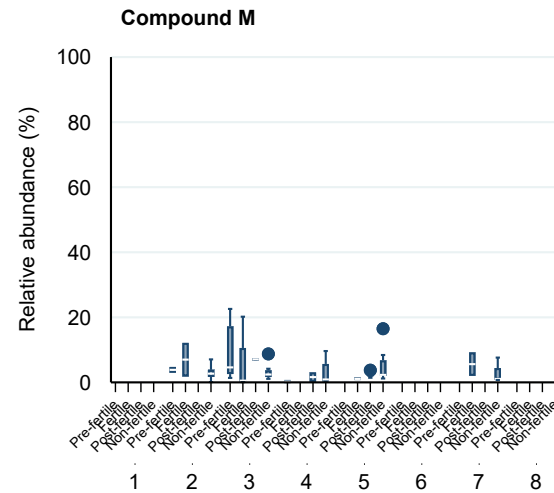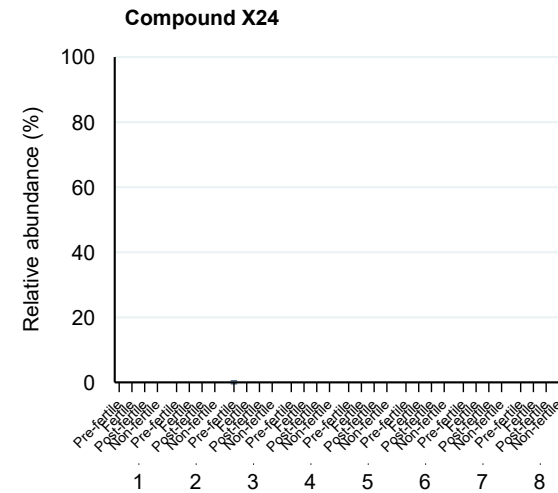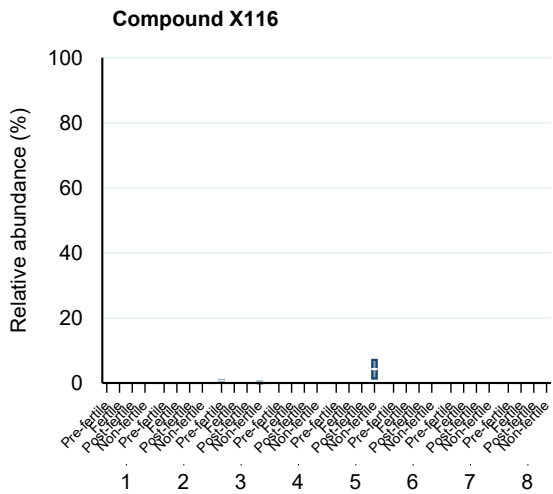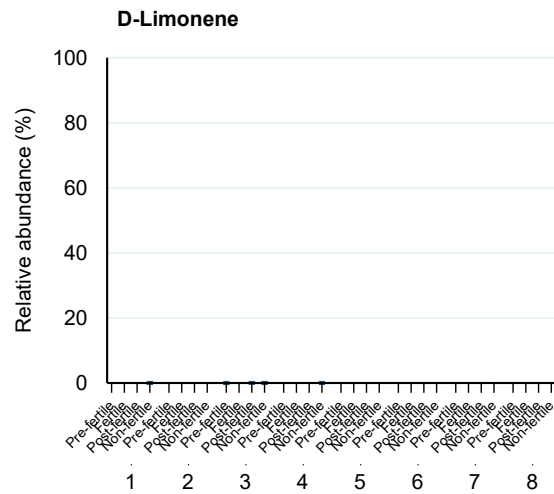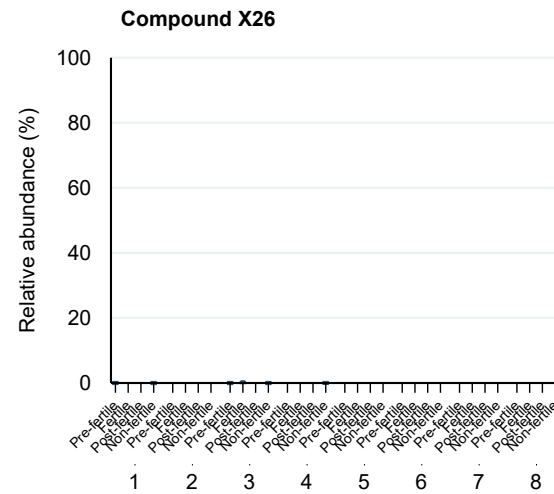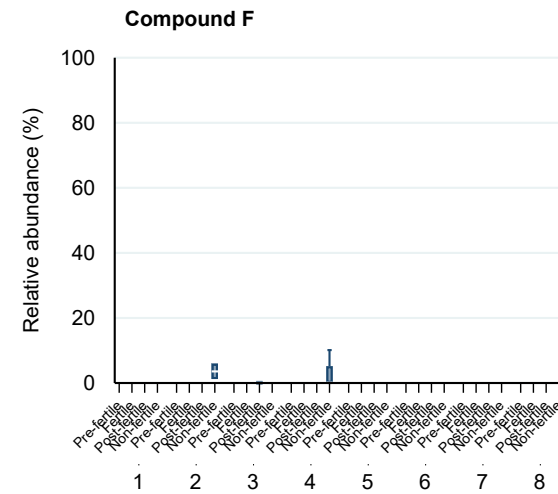

Supplement: Supplementary file 2 — Supplementary Figure S2. [file 41598_2021_87893_MOESM2_ESM.zip › Figures S2a-j/FigureS2d.pdf]

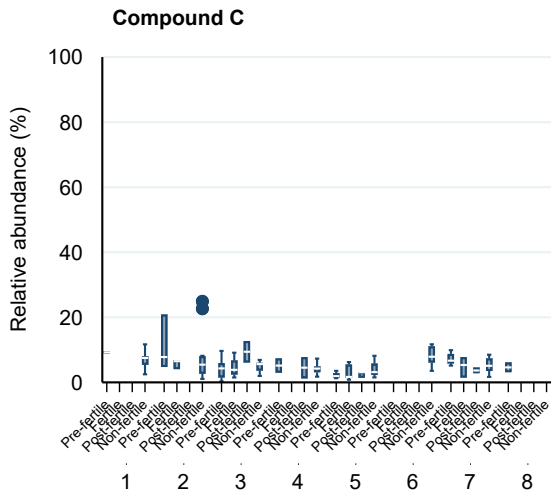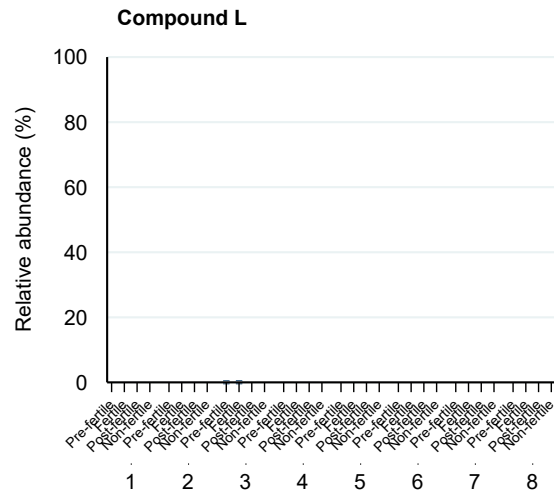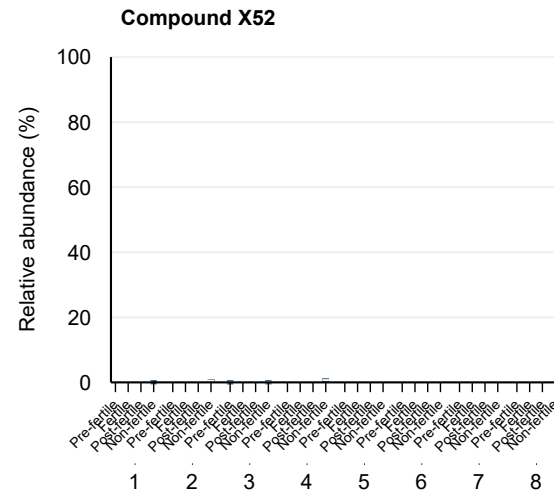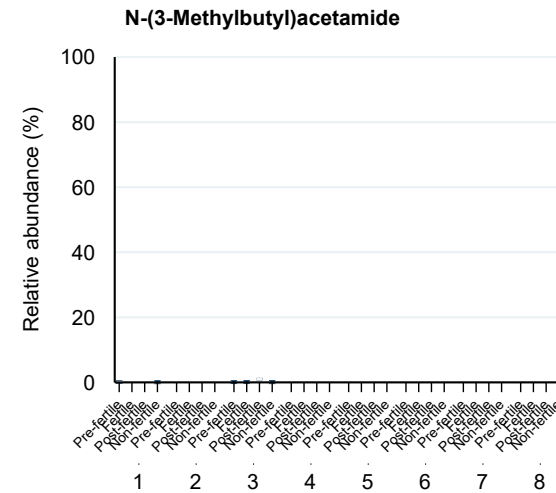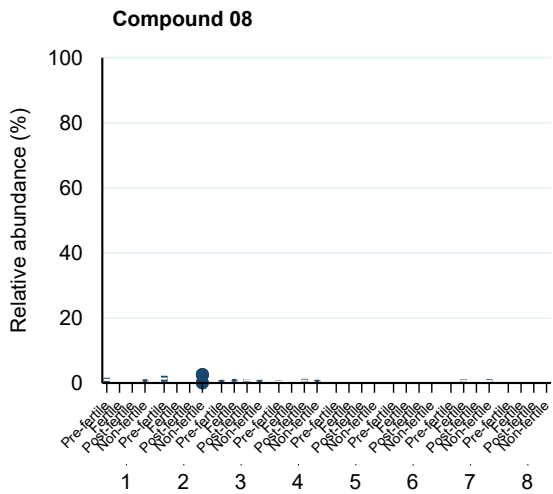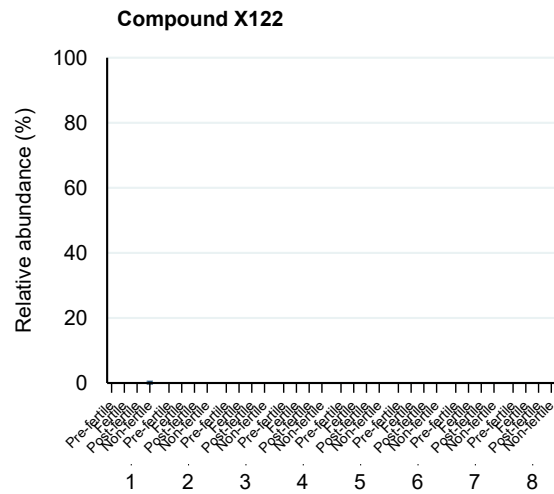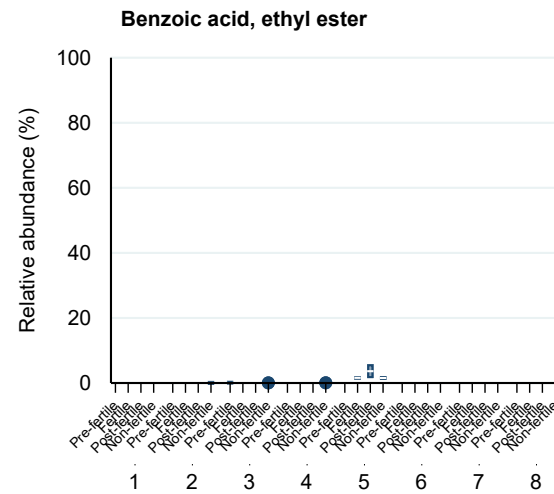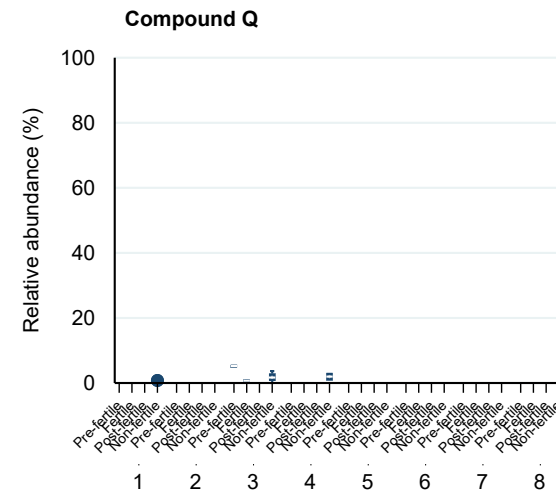

Supplement: Supplementary file 2 — Supplementary Figure S2. [file 41598_2021_87893_MOESM2_ESM.zip › Figures S2a-j/FigureS2f.pdf]

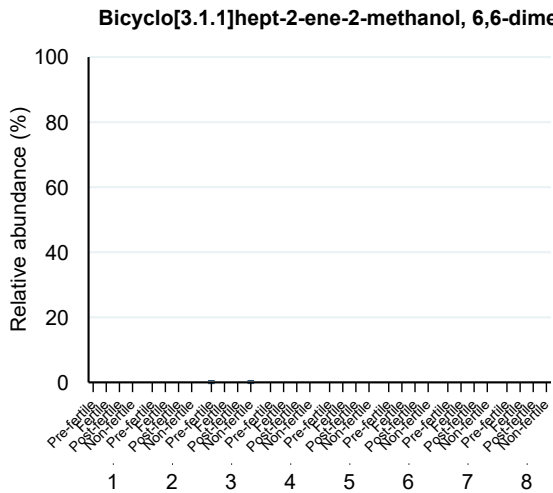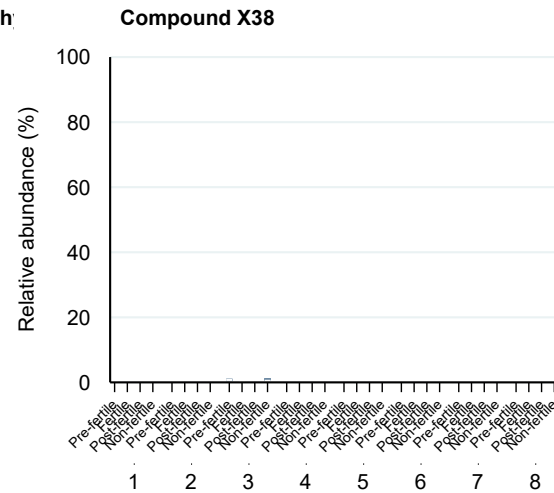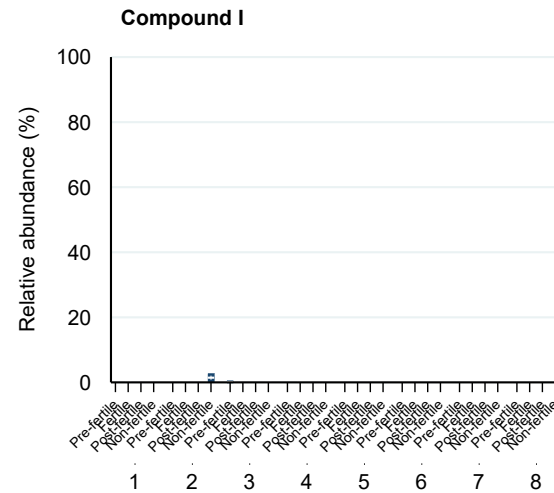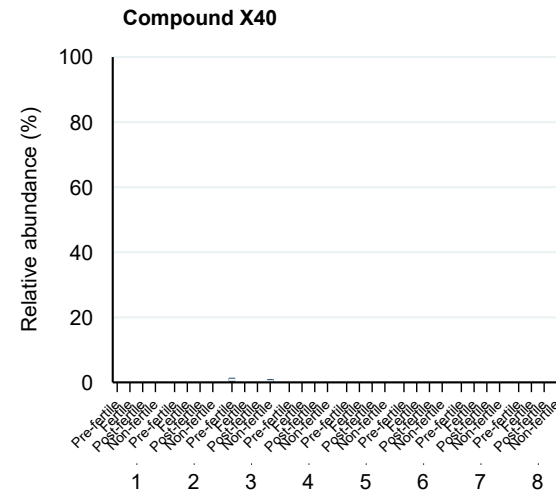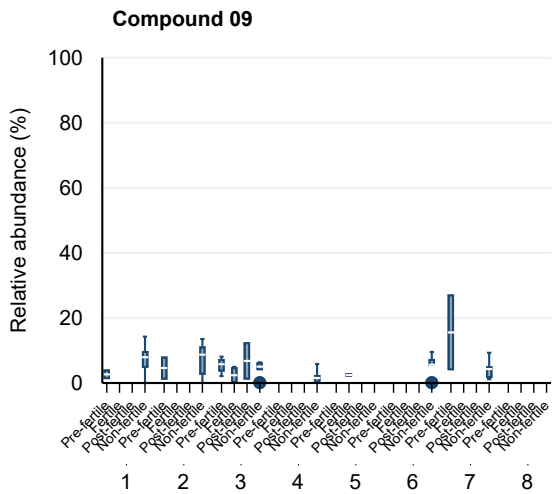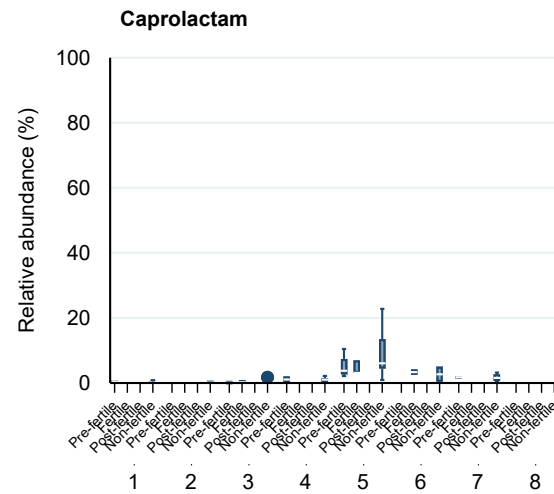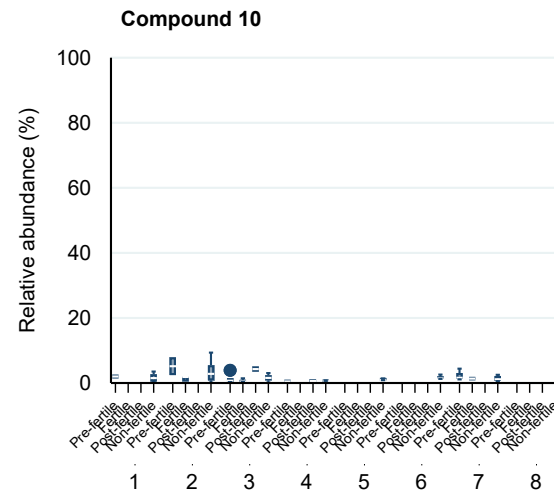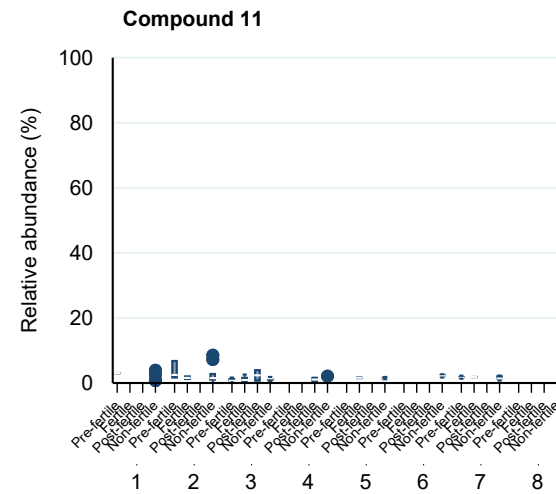

Supplement: Supplementary file 2 — Supplementary Figure S2. [file 41598_2021_87893_MOESM2_ESM.zip › Figures S2a-j/FigureS2g.pdf]

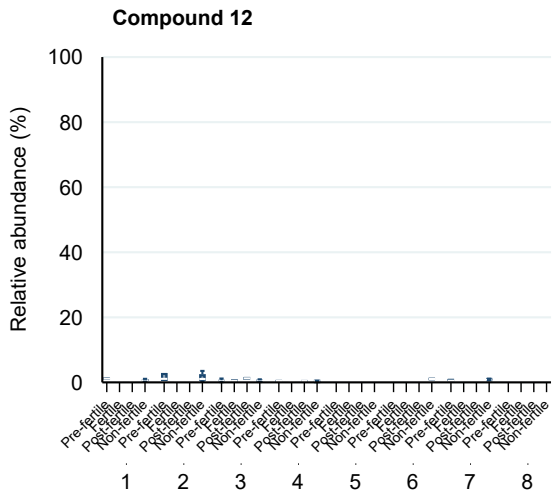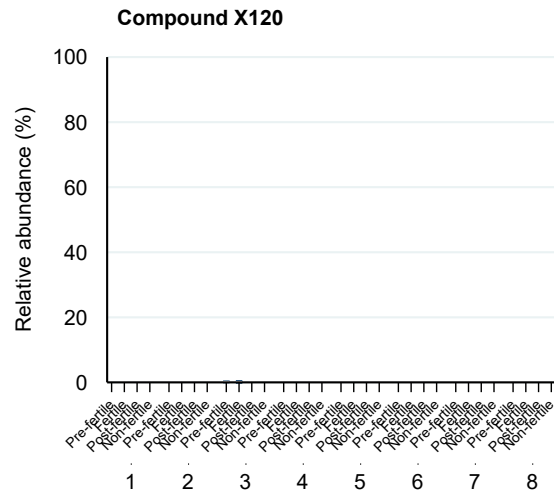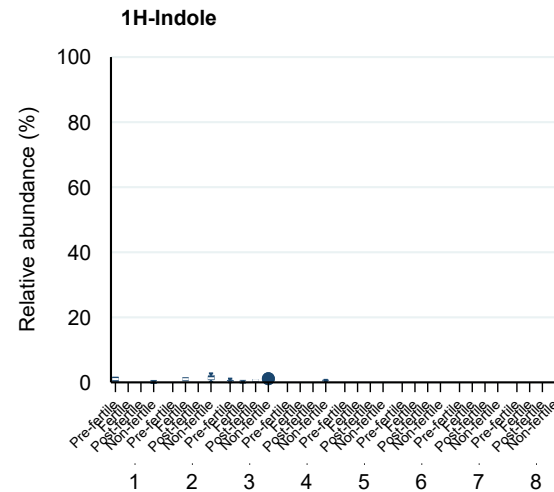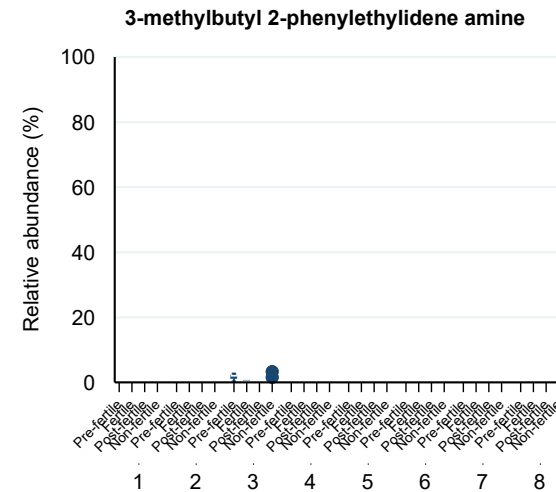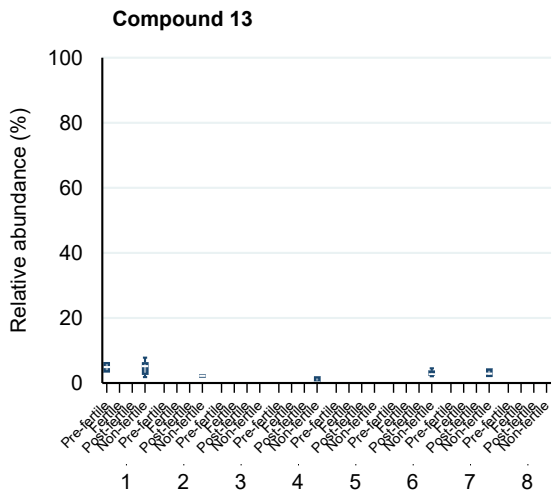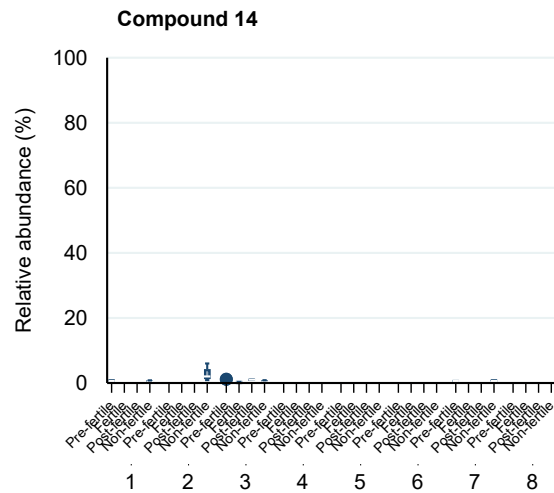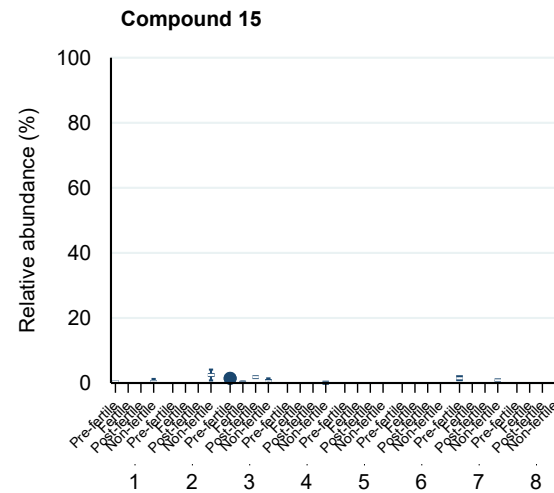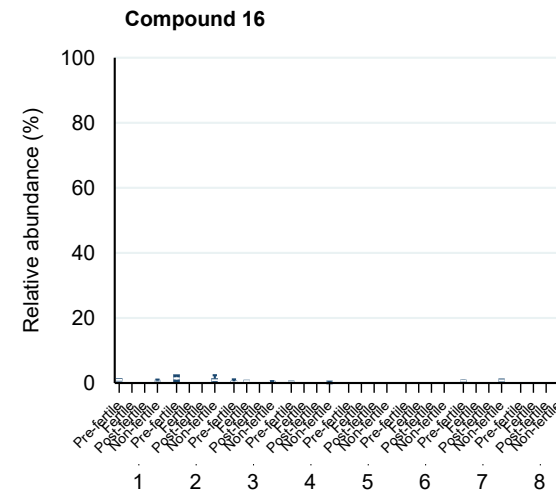

Supplement: Supplementary file 2 — Supplementary Figure S2. [file 41598_2021_87893_MOESM2_ESM.zip › Figures S2a-j/FigureS2h.pdf]

Compound X58

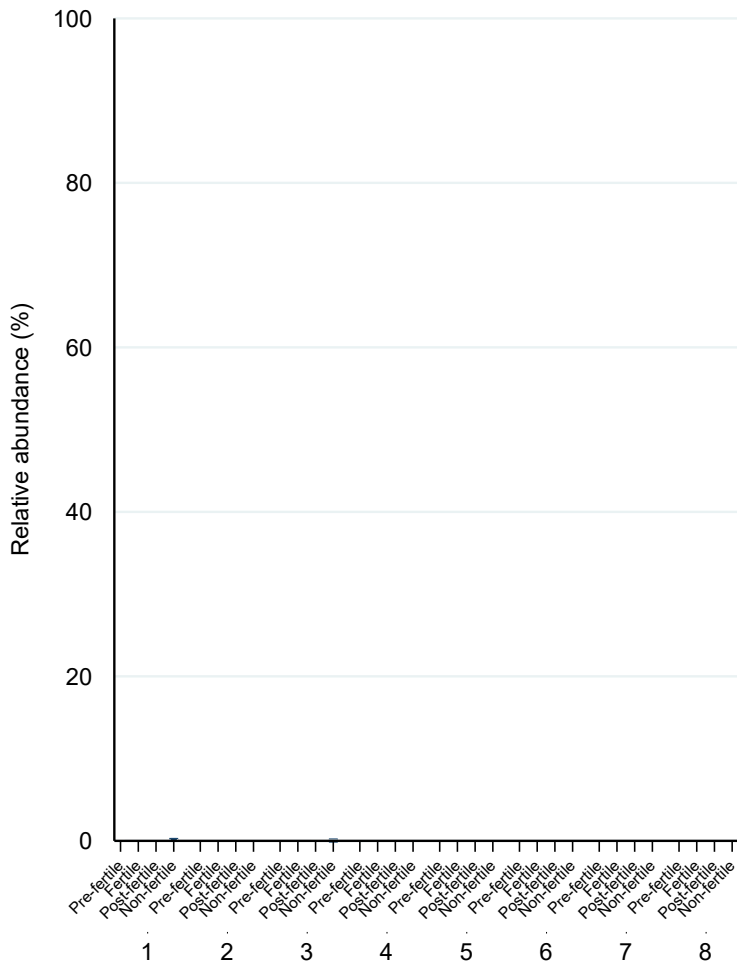

Compound 22

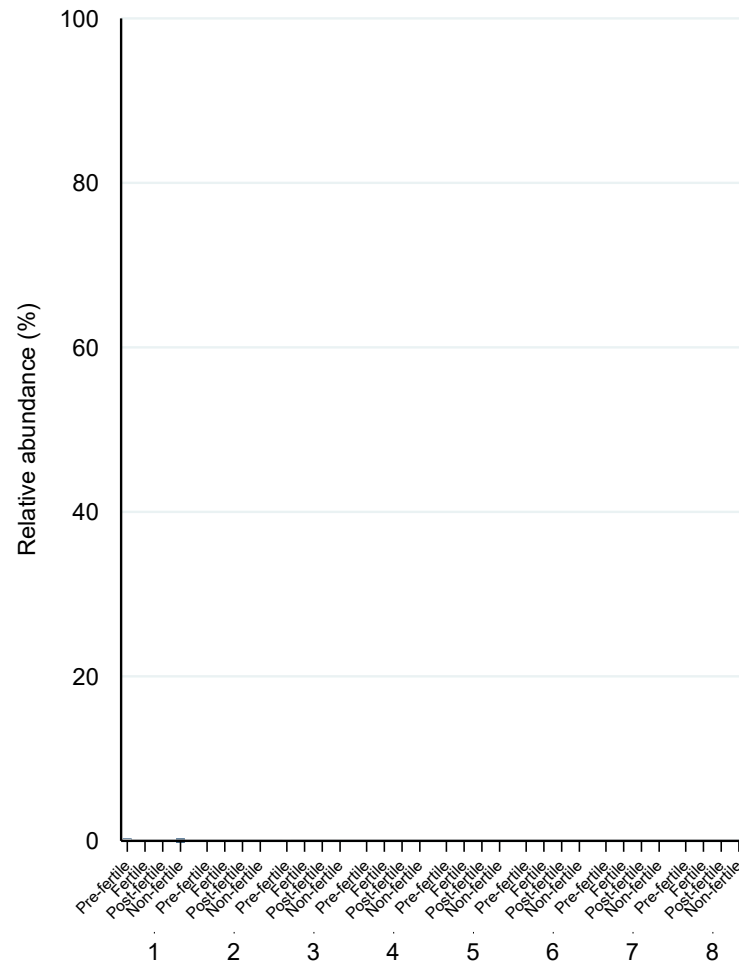

Unknown 05+Unknown 07

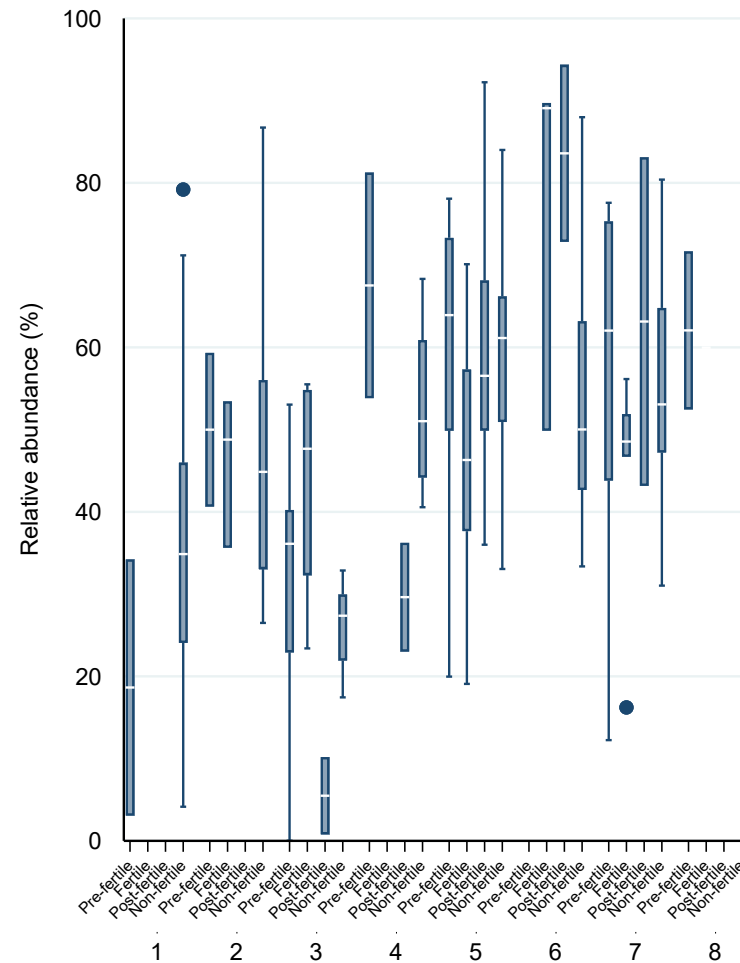

Supplement: Supplementary file 2 — Supplementary Figure S2. [file 41598_2021_87893_MOESM2_ESM.zip › Figures S2a-j/FigureS2j.pdf]
