# Supplementary material for: Olfactory signals and fertility in olive baboons
Source: Sci Rep. 2021 Apr 19;11:8506. doi: 10.1038/s41598-021-87893-6 (PMC8055877; doi:10.1038/s41598-021-87893-6)
Supplement: Supplementary file 2 — Supplementary Figure S2. [file FigureS2e.pdf › Figures S2a-j/FigureS2e.pdf]

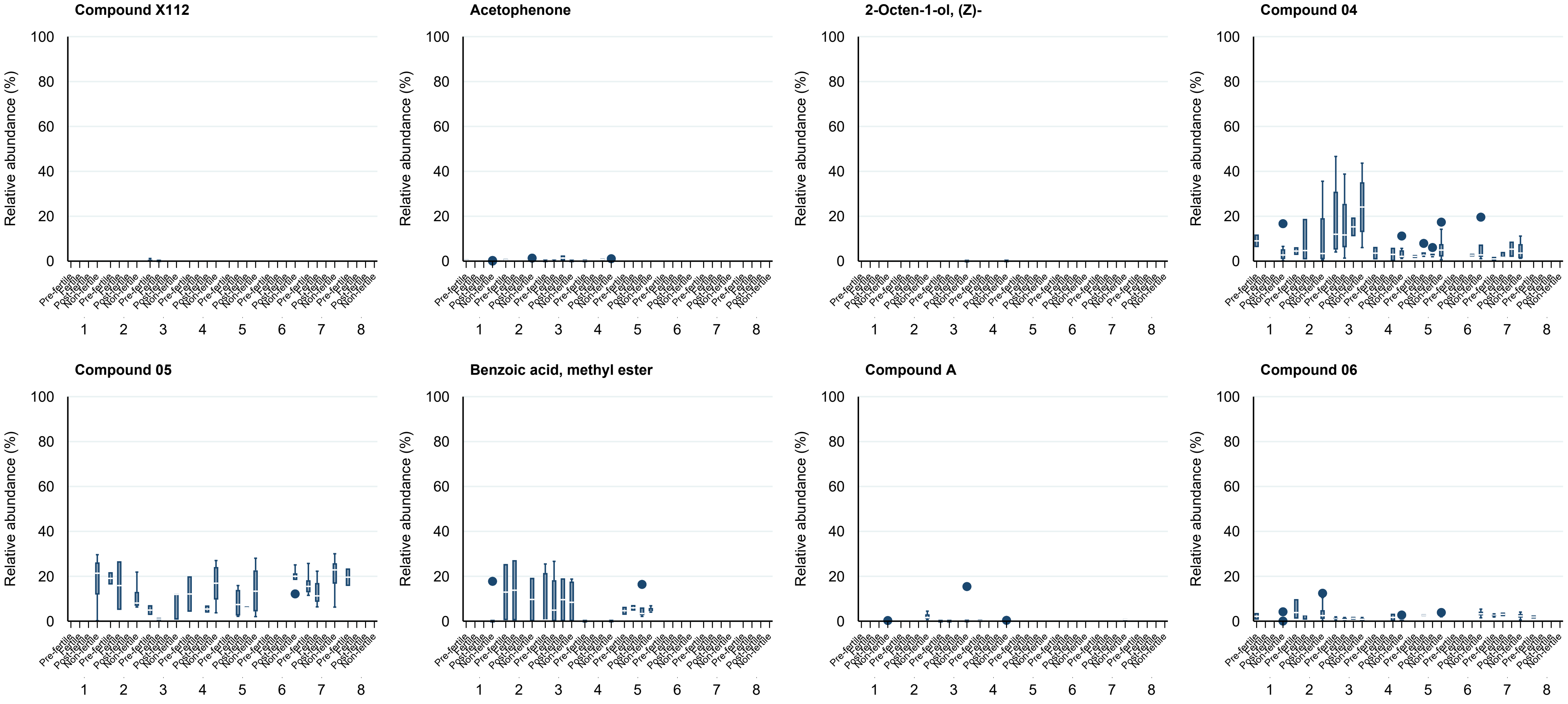
Compound X112
Acetophenone
2-Octen-1-ol, (Z)-
Compound 04
Compound 05
Benzoic acid, methyl ester
Compound A
Compound 06
Relative abundance (%)
0
20
40
60
80
100
Pre-fertile
Fertile
Post-fertile
Non-fertile
1
2
3
4
5
6
7
8
