# Supplementary material for: Olfactory signals and fertility in olive baboons
Source: Sci Rep. 2021 Apr 19;11:8506. doi: 10.1038/s41598-021-87893-6 (PMC8055877; doi:10.1038/s41598-021-87893-6)
Supplement: Supplementary file 2 — Supplementary Figure S2. [file FigureS2i.pdf › Figures S2a-j/FigureS2i.pdf]

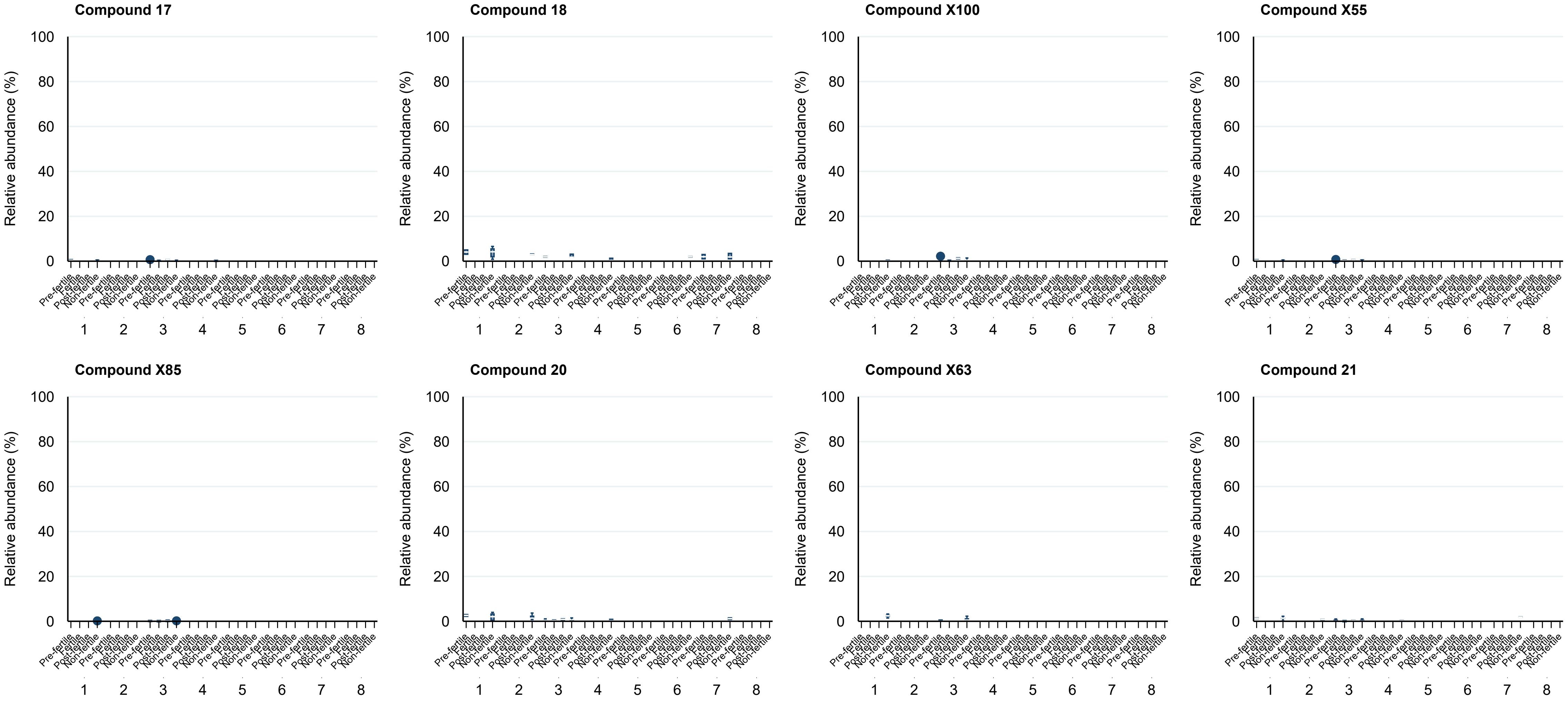

Compound 17
Compound 18
Compound X100
Compound X55
Compound X85
Compound 20
Compound X63
Compound 21
Relative abundance (%)
0
20
40
60
80
100
Pre-fertile
Fertile
Post-fertile
Non-fertile
1
2
3
4
5
6
7
8
